# Microneedles for controlled and sustained intraocular drug delivery

**DOI:** 10.1038/s41427-025-00614-7

**Published:** 2025-08-22

**Authors:** Junsang Lee, Jinheon Jeong, Van Phuc Nguyen, Seokkyoon Hong, Yannis M. Paulus, Chi Hwan Lee

**Affiliations:** 1 Weldon School of Biomedical Engineering, Purdue University, West Lafayette, IN 47907 USA; 2 Department of Ophthalmology, Wilmer Eye Institute, Johns Hopkins University, Baltimore, MD 21287 USA; 3 Department of Biomedical Engineering, Johns Hopkins University, Baltimore, MD 21287 USA; 4 School of Mechanical Engineering, Purdue University, West Lafayette, IN 47907 USA; 5 School of Materials Engineering, Purdue University, West Lafayette, IN 47907 USA; 6 Elmore School of Electrical and Computer Engineering, Purdue University, West Lafayette, IN 47907 USA; 7 Birck Nanotechnology Center, Purdue University, West Lafayette, IN 47907 USA

**Keywords:** Biomedical engineering, Biomaterials

## Abstract

Microneedles (MNs) have emerged as a promising technology for minimally invasive drug delivery, offering significant advantages in the treatment of ocular diseases. These miniaturized needles enable precise, localized drug delivery directly into specific tissues of the eye, such as the cornea, sclera, vitreous, or retina, while minimizing pain and discomfort. MNs can be fabricated from various biocompatible materials, including metals, silicon, and biodegradable polymers, making them highly adaptable to various clinical applications. Recent advancements in MN design include the integration of 3D printing technologies to create highly customized geometries for improved drug delivery precision, the use of smart materials that enable stimuli-responsive and sustained drug release, and the development of hybrid microneedles combining different polymers to enhance both mechanical strength and controlled drug release. These innovations have established MNs as a superior alternative to traditional methods like eye drops or intravitreal injections, which often face issues of limited bioavailability and patient compliance. This review summarizes the current state of research on MN-based ocular drug delivery, focusing on material developments, fabrication methods, drug release mechanisms, and implantation techniques. Future directions for MN technology in ophthalmology are also discussed, highlighting its potential to improve treatment outcomes for complex ocular diseases.

## Abstract (web summary)

Microneedles (MNs) are an innovative and minimally invasive approach to ocular drug delivery. Unlike traditional methods such as eye drops or injections, MNs can directly target specific eye tissues, including the cornea and retina, allowing for more effective treatment and improved patient comfort. This review highlights recent advances in MN technology, including 3D printing for customized geometries and the use of smart materials for sustained and controlled drug release. These innovations underscore the potential of MNs to transform treatment strategies for chronic eye diseases.

## Graphical abstract

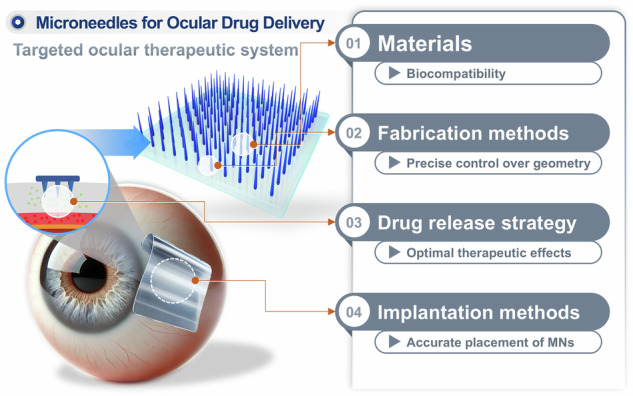

## Introduction

Microneedles (MNs) build upon the minimally invasive advantages of traditional hypodermic needles, extending their functionality into various forms. Unlike conventional needles, which typically penetrate deep into tissues, MNs consist of arrays of tiny needles, usually ranging from a few micrometers to about 900 µm in length. This design allows them to puncture the outermost layers of skin or targeted tissues with minimal discomfort and a reduced risk of tissue damage, while still delivering therapeutic agents effectively^1^. Their ability to enable drug delivery, biomarker sensing, and cell monitoring without causing significant pain or injury has made MNs an increasingly valuable tool in modern medicine^2–4^. As research progresses, MNs are gaining recognition for their potential to overcome the limitations of conventional delivery methods, especially in complex or delicate environments such as the eye^5,6^.

The delivery of drugs to the eye presents a unique set of challenges. The anatomy of the eye, with its multiple protective barriers such as the corneal epithelium and the blood-retinal barrier, makes it difficult to ensure that therapeutic agents reach the targeted area in effective concentrations^7,8^. Conventional methods of drug administration, such as eye drops or intravitreal injections, are often either too superficial or invasive. Eye drops, for instance, are quickly washed away by tears and blinking, leading to limited drug retention and requiring frequent dosing^9^. In contrast, intravitreal injections involve inserting a needle directly into the eye, posing risks such as infection, retinal detachment, and patient discomfort^10^. Additionally, many ocular conditions, such as age-related macular degeneration and diabetic retinopathy, require long-term management, making sustained and precise drug delivery particularly important^10^.

MNs offer a promising solution to the limitations of traditional ocular drug delivery. Their small size and minimally invasive nature allow them to deliver drugs directly to specific layers of the eye, such as the cornea or sclera, with much greater precision. By bypassing the natural barriers of the eye, MNs enable a more targeted approach, improving drug bioavailability and therapeutic efficacy^11^. Moreover, MNs can be engineered to provide sustained drug release, reducing the need for frequent interventions. For example, in treating conditions like macular degeneration, where frequent anti-vascular endothelial growth factor (VEGF) injections are typically required, MNs can deliver the drug in a controlled manner over time, maintaining therapeutic levels and minimizing the frequency of injections^12^. This capability not only improves patient compliance but also reduces the overall risks associated with repeated invasive procedures.

The design and application of MNs for intraocular drug delivery must take into account the delicate nature of ocular tissues and the diverse structural properties across different regions of the eye. Each region, from the cornea to the sclera and retina, presents unique challenges in terms of required penetration depth and drug distribution. For example, MNs designed for corneal delivery typically need to penetrate around 50 to 100 µm, while those targeting the deeper sclera or retina may require lengths between 200 and 500 µm^13^. Ensuring that the MNs are both long enough to reach the desired tissue but short enough to avoid deeper penetration is critical for safety and effectiveness. This specificity highlights the importance of tailoring MN designs to their target regions within the eye, ensuring that they meet the unique mechanical and pharmacokinetic demands of the tissue.

Another critical aspect of MNs design is material selection. Since MNs interact with sensitive ocular tissues, biocompatibility is paramount. Materials used in MNs must not provoke adverse immune responses or cause irritation. Biodegradable polymers such as polylactic acid (PLA) and hyaluronic acid (HA) are often favored because they dissolve gradually after delivering the drug, eliminating the need for removal and reducing the risk of long-term complications^14^. However, the mechanical properties of these materials must also be considered. MNs must be strong enough to penetrate the target tissue without bending or breaking, but also soft enough to avoid causing trauma to delicate ocular structures. The degradation rate of the MN material must also be matched to the desired drug release profile—fast enough to release the therapeutic agent in a timely manner but slow enough to sustain drug levels over the required treatment period^15^.

The controlled release of drugs is one of the most important aspects of MN technology for intraocular drug delivery. MNs can be engineered to deliver drugs either immediately upon insertion or gradually over time, depending on the needs of the patient and the condition being treated. For chronic diseases such as glaucoma, where consistent therapeutic levels must be maintained, MNs with sustained release systems offer significant advantages^16^. Various strategies can be employed to achieve this, such as using biodegradable coatings that dissolve at predetermined rates or incorporating reservoirs within the needle structure that release drugs as the material degrades^8,15,17^. These controlled release systems help maintain therapeutic drug concentrations in the eye for extended periods, reducing the need for frequent dosing and improving treatment outcomes. Moreover, for more complex treatments requiring the delivery of multiple drugs, MNs can be designed with different layers or compartments that release drugs sequentially, ensuring precise timing and dosage of therapeutic agents^18^.

Recent advances in MN technology have opened new possibilities for intraocular drug delivery. Innovations in both material science and fabrication techniques have enabled the creation of more complex MN systems capable of addressing a broader range of therapeutic needs. For instance, the use of smart materials that respond to environmental triggers—such as changes in pH or temperature—has improved the precision of drug delivery^19–21^. Additionally, advances in fabrication techniques, including micro-molding and 3D printing, allow for the creation of multi-layered or hollow MNs capable of delivering larger doses of drugs or accommodating more complex release profiles^5,22^. These innovations have the potential to revolutionize the treatment of ocular diseases, offering more effective and less invasive therapeutic options for patients.

**Fig. 1:**
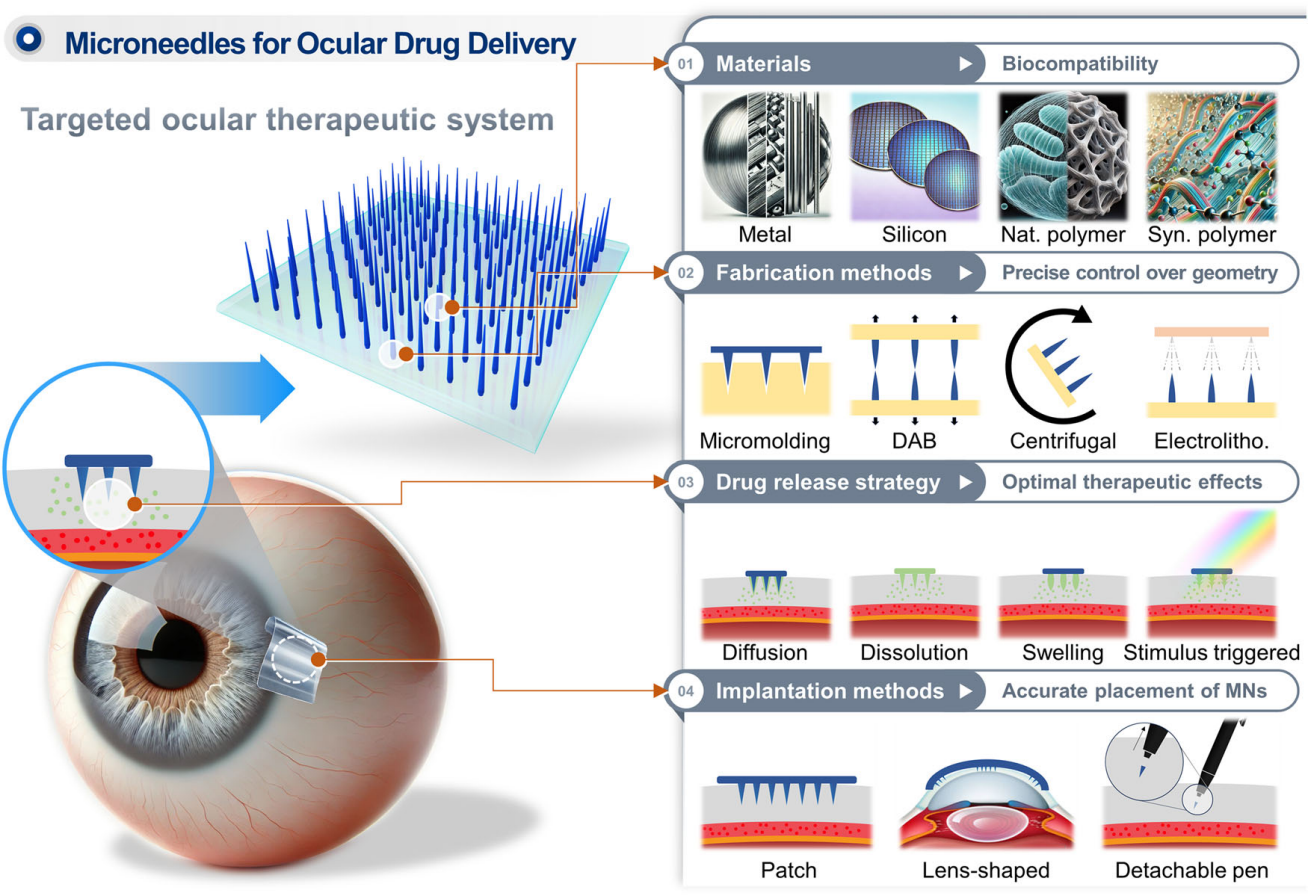
Schematic overview of MNs for ocular drug delivery.

In this review, we aim to provide a comprehensive overview of MNs as they apply to ocular drug delivery, focusing on four critical aspects: materials selection, fabrication methods, drug release mechanisms, and implantation methods. Each of these elements plays a crucial role in determining the effectiveness of MNs for treating ocular diseases. Figure 1 illustrates a MN patch applied to the eye, highlighting the MN’ structure and the four core areas that will be discussed in this paper. By exploring the latest research and developments in these areas, this review seeks to offer valuable insights into how MNs can be further optimized for clinical use, ultimately contributing to the advancement of safer and more effective treatments for a wide range of ocular conditions.

In this review, we aim to provide a comprehensive overview of MNs as they apply to ocular drug delivery, focusing on four critical aspects: materials selection, fabrication methods, drug release mechanisms, and implantation methods. Each of these elements plays a crucial role in determining the effectiveness of MNs for treating ocular diseases. Figure 1 illustrates a MN patch applied to the eye, highlighting the key aspects covered in this review. To develop effective MN systems for ocular drug delivery, we focus on four core areas: material selection, fabrication methods, drug release strategies, and implantation techniques. Materials such as metals, silicon, and polymers are essential for balancing biocompatibility and mechanical properties, while advanced fabrication techniques ensure precise control over MN geometry. Effective drug release strategies, including diffusion and stimulus-triggered release, are critical for achieving sustained therapeutic effects, and implantation methods like patch-based or lens-shaped designs ensure accurate delivery to ocular tissues. This figure provides an overview of these interconnected elements, demonstrating how each contributes to advancing MN-based ocular therapies. By exploring the latest research and developments in these areas, this review seeks to offer valuable insights into how MNs can be further optimized for clinical use, ultimately contributing to the advancement of safer and more effective treatments for a wide range of ocular conditions.

## Materials of MNs for Intraocular Drug Delivery

Material selection plays a pivotal role in the design of MNs for ocular applications, where precision, biocompatibility, and controlled drug release are essential. The eye’s complex anatomy, coupled with its slow regeneration and liquid-based environments, makes it particularly sensitive to therapeutic interventions. Designing MNs for ocular drug delivery requires navigating these challenges to ensure minimal tissue damage, effective drug absorption, and sustained therapeutic effects. Additionally, the risk of vision obstruction, irritation, or drug loss due to blinking adds another layer of complexity to the design process. Unlike other areas of the body, where MNs primarily interact with skin or muscle, ocular applications demand materials that can maintain mechanical integrity upon insertion while gradually degrading or dissolving without causing adverse effects. Thus, this section will explore key considerations for MN materials, focusing on factors such as biocompatibility, biodegradability, and mechanical stability, as well as how these properties can be tailored to meet the specific demands of ocular drug delivery. Each material discussed has been chosen for its unique ability to address the challenges posed by the eye’s intricate structure, ensuring both efficacy and safety in long-term treatment strategies.

### Metal Based MNs

Metal MNs were among the first materials used for MN applications due to their exceptional mechanical strength, durability, and biocompatibility. Before the development of polymer-based MNs, metals such as stainless steel and titanium were favored for their rigidity, which allowed them to easily penetrate tissues without bending or breaking. This was particularly critical in early applications where precision and reliable insertion were necessary to ensure that MNs could deliver drugs or vaccines effectively. Stainless steel is highly resistant to corrosion, making it particularly suitable for repeated exposure to the liquid environments of the eye^23,24^. Titanium, on the other hand, offers a balance of durability and flexibility, which is critical for designing MNs that can adapt to the eye’s varying structural properties without breaking^25,26^. Ensuring the long-term biocompatibility of these materials is crucial, especially in the delicate ocular environment. Both stainless steel and titanium are commonly used in Food and Drug Administration (FDA)-approved medical devices, reflecting their safety in clinical applications.

**Fig. 2:**
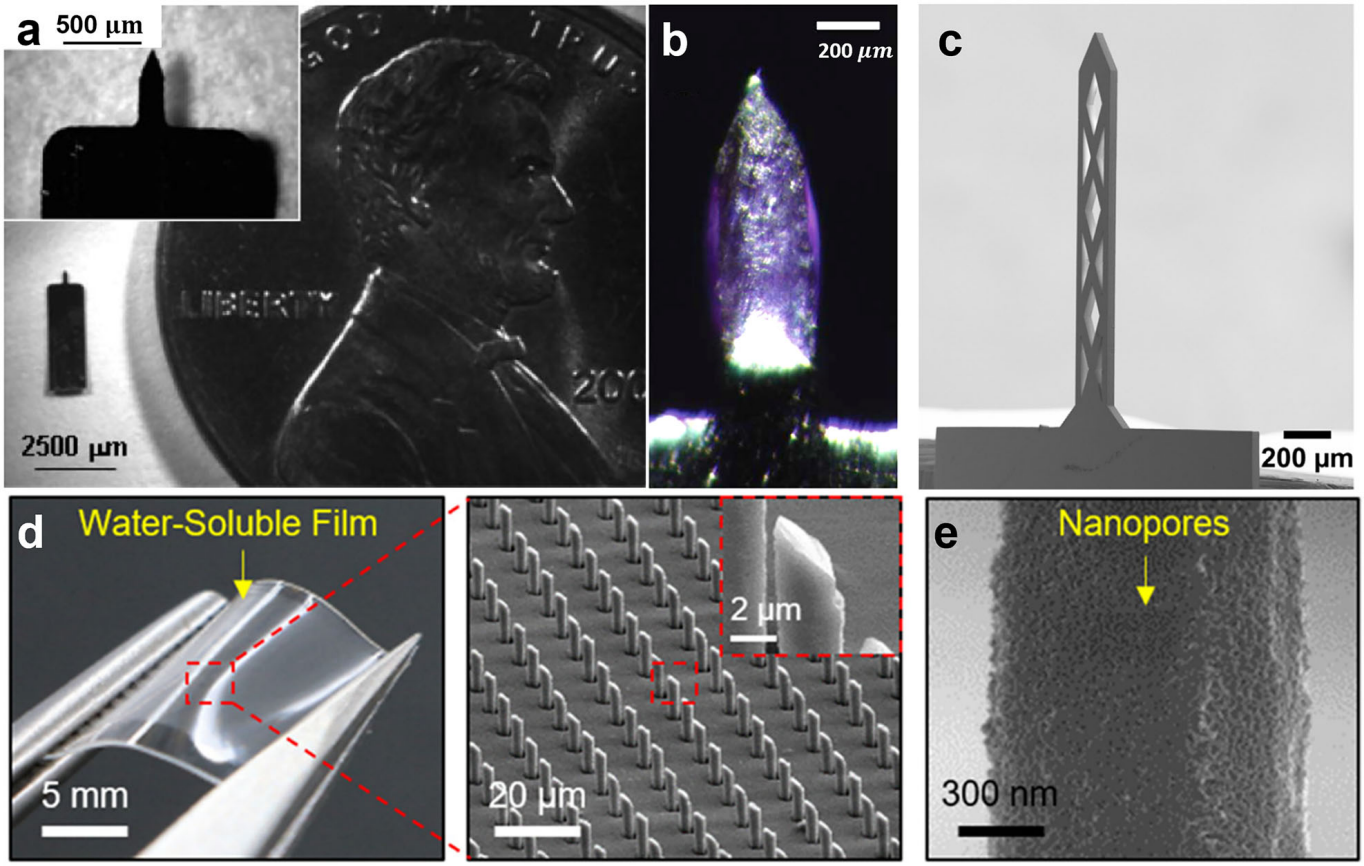
Metals and silicon-based MNs. **a** Optical microscope (OM) images of a single stainless-steel MN for intrascleral and intracorneal drug delivery. Reproduced with permission from ref. ^27^. **b** OM of stainless-steel MN coated with bevacizumab. Reproduced with permission from ref. ^28^. **c** Scanning electron microscope (SEM) image of fenestrated titanium MN. Reproduced with permission from ref. ^26,29^. **d** OM of the porous Si needles on a PVA film. Magnified image highlighting the sharpened angular tip of Si needles. Reproduced with permission from ref. ^34^. **e** SEM image of the porous Si needle surface with nanopores. Reproduced with permission from ref. ^34^.

The fabrication of metal MNs also benefited from advanced techniques like laser cutting and deep reactive ion etching (DRIE), which allowed for highly controlled geometries. Jiang et al. utilized laser cutting to fabricate stainless steel MNs with precise dimensions of 400 µm in length, 150 µm in width, and 75 µm in thickness, as shown in Fig. 2(a)^27^. These MNs were coated with bevacizumab, a monoclonal antibody, using a dip-coating method, allowing for highly targeted drug delivery directly into the corneal stroma without crossing into the endothelium. This targeted approach demonstrated a significant reduction in corneal neovascularization in rabbit models, with lower doses of bevacizumab compared to traditional treatments. The reduced drug dosage, combined with high efficacy, showcases the potential of metal MNs for precise, localized drug delivery in ocular treatments.

Kim et al. extended this application by using stainless steel MNs coated with bevacizumab for intra-stromal delivery, specifically targeting corneal neovascularization, as depicted in Fig. 2b^28^. This method provided precise, localized drug delivery directly into the corneal stroma and significantly reduced the growth of blood vessels in the cornea, with minimal systemic drug exposure. Compared to traditional methods like topical eye drops or subconjunctival injections, stainless steel MNs required much smaller drug doses while achieving similar therapeutic effects. The ability to reduce drug doses without compromising efficacy is a significant advantage of metal MNs in treating ocular conditions, offering both patient safety and cost efficiency.

In addition to stainless steel, titanium has been explored for its durability and flexibility in MN design. Khandan et al. fabricated titanium-based fenestrated MNs using a DRIE process, producing MNs with lengths ranging from 500 to 1500 µm and widths of 50 to 200 µm, as seen in Fig. 2(c)^26,29^. The fenestrated design allowed for increased drug-loading capacity, providing up to five times more drug storage compared to solid MNs of the same size. These fenestrations acted as drug reservoirs, enabling passive ocular drug delivery through diffusive transport. The titanium MNs were coated with Rhodamine B using a dip-coating method, employing polyvinylpyrrolidone (PVP) as a viscosity enhancer and Lutrol F-68 as a surfactant. This design allowed for controlled release of the drug over time upon insertion into corneal tissue. Tests conducted on ex vivo rabbit models showed efficient drug delivery, without causing damage to either the MNs or the surrounding tissue.

While polymer-based MNs are favored for their biodegradability, metal MNs provide superior mechanical strength and stability, making them ideal for applications that require precise and repeated tissue penetration, such as intraocular drug delivery. Whether designed for highly precise, localized treatment or for maximizing drug storage through innovative structures like fenestrations, metal MNs continue to provide reliable, durable solutions for ocular drug delivery. Their strength, coupled with their biocompatibility and ability to support controlled drug release, makes them ideal candidates for long-term therapeutic applications.

### Silicon Based MNs

Compared to metals like stainless steel or polymers, silicon (Si) offers higher precision due to its well-established microfabrication techniques from the semiconductor industry. This makes it ideal for applications where extremely fine geometries and high structural integrity are essential. Its mechanical strength, biocompatibility, and established fabrication techniques allow for the creation of highly precise, robust MNs. Si MNs are generally strong enough to penetrate biological tissues, including sensitive areas like the cornea or sclera, with minimal risk of breakage, making them suitable for ocular applications. Additionally, Si is non-toxic and suitable for long-term biomedical use, ensuring safety and reliability in drug delivery systems. However, despite its overall strength, Si does possess inherent brittleness, which could pose a risk of breakage in specific circumstances, particularly under high-stress conditions^30^. To mitigate this risk, fabrication techniques such as reactive ion etching (RIE) and anisotropic etching are used to create geometrically optimized structures^31–33^. Moreover, integrating soft substrates, like PVA and PDMS, helps further reduce the risk of needle breakage during application, combining the precision of Si with the flexibility of polymers for safer and more reliable drug delivery^34–36^.

One of the key advantages of Si MNs is their precision. Kim et al. used a combination of photolithography, dry and wet etching, and metal-assisted chemical etching (MACE) to fabricate Si MNs with tip diameters as small as 150 nm and base diameters ranging from 2 to 4 μm^34^. These MNs, shown in Fig. 2d, were transferred onto a water-soluble polyvinyl alcohol (PVA) film for support. Upon insertion into tissue, the PVA film dissolved within one minute, leaving the MNs embedded in the tissue. This design enabled sustained drug release through nanopores on the needle surface, which were controlled by varying the MACE time, thereby allowing for greater drug-loading capacity and prolonged drug release^34^, as seen in Fig. 2e.

The versatility of Si extends to different delivery approaches. Park et al. demonstrated the use of Si nanoneedles (Si NNs) integrated into a tear-soluble contact lens for ocular drug delivery. The NNs, with base diameters controlled to 900 nm and lengths of up to 60 μm, could penetrate the corneal epithelium without causing significant corneal or ocular damage^36^. Once in place, the PVA-based contact lens dissolved in tear fluid within a minute, releasing the embedded NNs into the cornea. The NNs gradually degraded over time, providing sustained drug delivery for months. The surface porosity, which could be adjusted during fabrication, further enhanced the drug-loading capacity and allowed for precise control over the release rate. Covalent functionalization with drugs such as anti-VEGF ensured stable and targeted release, demonstrating effectiveness in treating conditions like corneal neovascularization in a rabbit model.

In both examples, Si’s mechanical robustness and biocompatibility were key to the success of these drug delivery systems. Whether used in combination with dissolvable substrates or directly inserted into tissue, Si MNs provide a stable platform for controlled and sustained drug release. These properties make Si an ideal material for ocular drug delivery systems, particularly in applications requiring precise, minimally invasive treatments over extended periods.

### Poly(lactic-co-glycolic acid) and poly(lactic acid) based MNs

Synthetic biodegradable polymers, such as poly(lactic-co-glycolic acid) (PLGA) and PLA, have emerged as key materials in drug delivery systems due to their biocompatibility, gradual degradation, and ability to safely break down into non-toxic byproducts^37^. Their biocompatibility is particularly essential in sensitive applications like ocular drug delivery, where minimizing immune responses and ensuring a controlled release of therapeutic agents is crucial^38^. The gradual degradation of these polymers makes them ideal for sustained drug release, providing long-term therapeutic effects without the need for frequent dosing. This makes PLGA and PLA especially suitable for conditions like chronic eye diseases, where consistent and localized drug administration is necessary to maintain efficacy.

Both PLGA and PLA offer tunable degradation profiles, allowing researchers to modify their molecular composition or adjust the fabrication process to achieve desired drug release rates. For example, by varying the ratio of lactic acid to glycolic acid in PLGA, researchers can control how quickly the polymer degrades^39,40^. A higher glycolic acid content generally results in faster degradation, while increasing the lactic acid content slows the degradation rate. PLGA typically degrades over a few weeks to months^39^, while PLA offers an even slower degradation rate, making it well-suited for applications requiring long-term drug release^41^. As the MNs gradually degrade, they provide a steady and controlled release of encapsulated drugs, further enhancing their therapeutic value in treating conditions affecting the cornea, sclera, vitreous, retina, and other ocular regions.

**Fig. 3:**
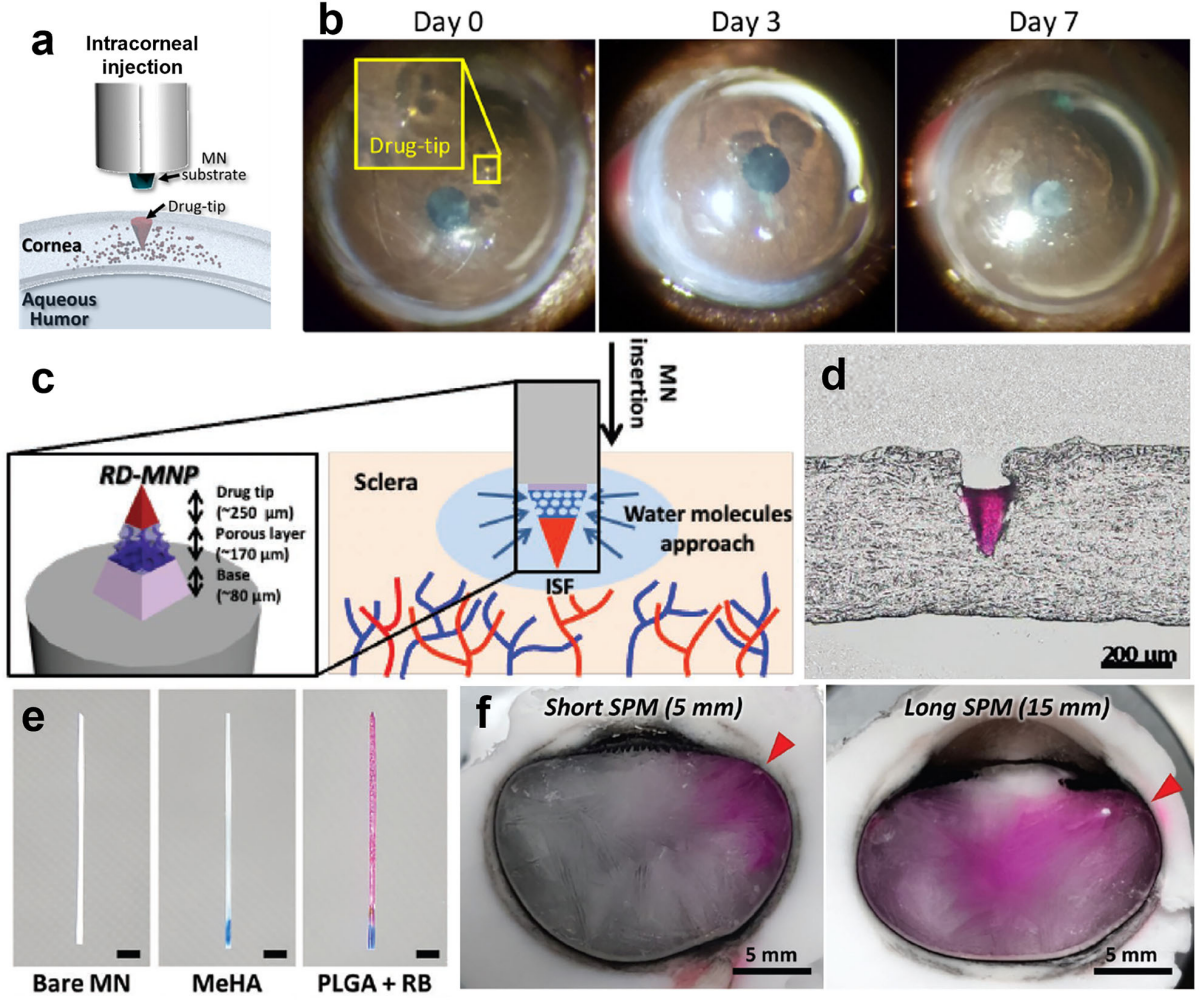
PLA and PLGA-Based MNs. **a** Schematic of detachable MN. Reproduced with permission from ref. ^42^. **b** Microscope images of cornea after insertion of detachable MN. Reproduced with permission from ref. ^42^. **c** Schematic structure and concept of MN with sacrificial layer. Reproduced with permission from ref. ^43^. **d** Cryo-sectioned images of MN penetrated the scleral tissue. Reproduced with permission from ref. ^43^. **e** OM of bare, Methacrylated hyaluronic acid and PLGA with Rhodamine coated MN. Reproduced with permission from ref. ^44^. **f** OM of drug distribution depending on the MNs length of 5 mm (left) 15 mm (right). Reproduced with permission from ref. ^44^.

In recent studies, PLGA-based MNs have been leveraged for targeted ocular applications. An intracorneal injection MN system for sustained drug delivery was developed, particularly targeting Acanthamoeba keratitis as shown in Fig. 3a^42^. These MNs featured a detachable PLGA-based drug tip that dissolved gradually within the corneal tissue, providing a controlled release of the drug over time. The drug-loaded MN successfully penetrated the cornea, with the PLGA tip remaining in place and dissolving over a period of days, as confirmed by histological observations in Fig. 3b. The in vitro drug release profile indicated that 93% of the drug was released within the first four days, with complete dissolution occurring by day nine, demonstrating the controlled and sustained release of the encapsulated therapeutic agent. A similar study from the same research group applied PLGA-based MNs to target the sclera, achieving rapidly detachable MN pen using a sacrificial layer^43^. A highly soluble porous structured PVA and PVP layer dissolved as water molecules contacted it as shown in Fig. 3c. The pre-molded PLGA needle tip, embedded with a magenta-colored fluorescent dye, successfully penetrated the scleral tissue, as observed in Fig. 3d.

The gradual dissolution of the PLGA material provided a steady drug release profile, supporting the use of these MNs for posterior segment diseases. Intravitreal drug delivery has been demonstrated using MNs fabricated with a PLA base and a methacrylated hyaluronic acid (MeHA) -coated tip for self-plugging upon insertion^44^. The remaining portion of the needle was coated with PLGA and Rhodamine B as shown in Fig. 3e, ensuring sustained delivery of the drug over 24 hours. By varying the length of the MNs, as demonstrated in Fig. 3f, researchers were able to control the depth of drug delivery and fine-tune the drug dosage. This length-based customization, coupled with the biodegradable properties of PLGA and PLA, offers a highly adaptable solution for treating a range of ocular conditions.

One of the key challenges with using PLGA and PLA for drug delivery is their slow degradation rates, which can be a disadvantage in applications requiring immediate or rapid drug release. For instance, while PLGA and PLA provide controlled, sustained drug release over time, this characteristic can delay the onset of drug action in acute treatments where fast therapeutic responses are needed, such as in ocular infections^39,40^. Furthermore, the degradation of these polymers often results in inconsistent drug release profiles, where an initial burst of the drug is followed by a slower, more gradual release. This inconsistency can lead to challenges in achieving precise dosage control and may affect therapeutic efficacy in some cases^39^. Additionally, due to their hydrophobic nature, the release rates of drugs from PLGA and PLA are influenced by environmental factors such as pH and temperature, which further complicates their use in certain sensitive tissues^40^. To address these limitations, combination and substitution with rapidly dissolving materials are being explored as alternatives, offering quicker degradation and more consistent release for acute treatments.

### Hyaluronic Acid based MNs

HA is a naturally occurring biopolymer widely used in drug delivery systems due to its excellent biocompatibility, biodegradability, and hydrophilic properties^6,17,45,46^. As a naturally present glycosaminoglycan found in connective, epithelial, and neural tissues^45^, HA plays a crucial role in maintaining tissue hydration, making it highly suitable for ocular applications. In drug delivery, its high-water retention ability allows for enhanced bioavailability and sustained release of drugs over time^47^. This property is particularly beneficial in ophthalmic formulations, where moisture retention and controlled drug release are essential. HA excels in applications requiring enhanced moisture retention and minimal irritation^6^. This makes HA particularly advantageous for sensitive ocular tissues like the cornea, where minimizing friction and irritation is critical^45^. Its inherent lubricating properties^45^ also make it an ideal candidate for ocular drug delivery as it minimizes friction during MN insertion and reduces the risk of irritation in sensitive tissues such as the cornea and sclera^48^. At the same time, self-sealing properties of HA-coated needles for intravitreal injections was confirmed by Eom et al., emphasizing HA’s role in preventing vitreous humor and drug reflux through the needle passage^48^. This ability to maintain a tight seal post-injection significantly reduces the risk of infection and drug leakage, making it an essential feature for ocular applications.

**Fig. 4:**
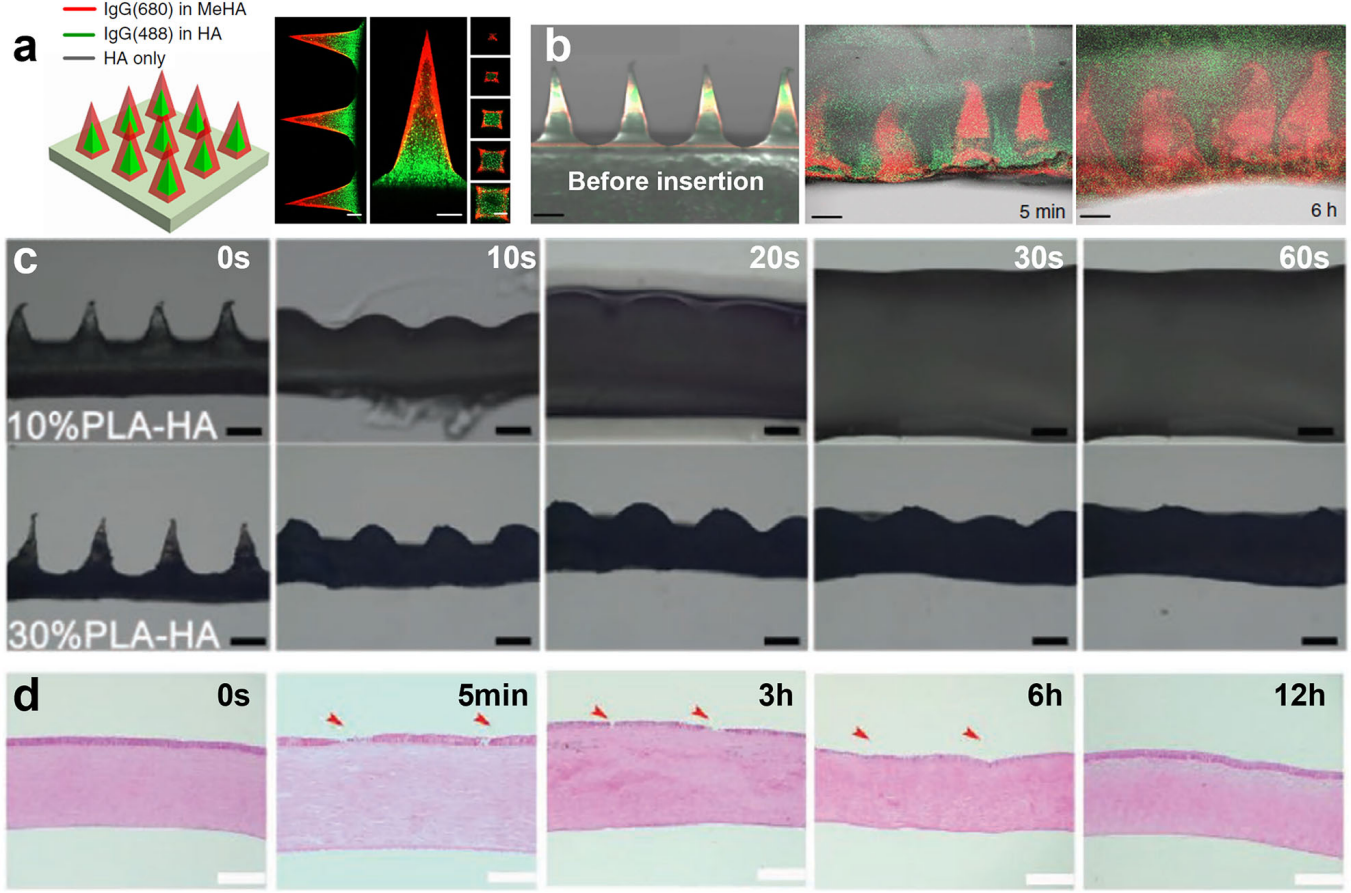
HA-Based MNs. **a** Schematic of DL-MNs using MeHA and HA (left) Confocal image DL-MNs loading immunoglobulin G colored with Alexa Fluor 680 (red) and 488 (green) Scale bar: 100 μm (right). Reproduced with permission from ref. ^18^. **b** Drug release distribution images of DL-MNs in agarose hydrogel. Reproduced with permission from ref. ^18^. **c** Time-dependent dissolution of MNs with different PLA contents (Scale bar: 200 μm). Reproduced with permission from ref. ^51^. **d** H&E images of the cornea before and after insertion of 30% PLA MNs (Scale bar: 200 μm). Reproduced with permission from ref. ^51^.

In terms of drug release, HA can be crosslinked or chemically modified (e.g., MeHA) to form hydrogels that provide controlled and prolonged release of drugs^17,18,49^. A higher crosslinking density typically slows the degradation rate, leading to a more prolonged drug release as the tightly crosslinked network takes longer to break down. Conversely, lower crosslinking density results in faster degradation and quicker release of the encapsulated drugs. Moreover, HA-based systems can be fine-tuned to achieve desired release profiles by adjusting the molecular weight of the HA^49,50^. Higher molecular weight HA forms more stable hydrogels, providing slower and more sustained drug release, while lower molecular weight HA degrades faster, allowing for a quicker release profile. Double-layered MN (DL-MN) system, as shown in Fig. 4(a), effectively utilizing the difference between HA and MeHA for corneal neovascularization treatment was proposed^18^. Sequential micromolding method with MeHA and HA allows biphasic drug release system, as depicted in Fig. 4a, presenting two different drugs can be loaded separately in the outer and inner layers. Drug loaded in inner layer (green) released faster as HA dissolved rapidly, while the drug loaded in outer layer (red) showed sustained release as MeHA gradually discharge over hours as shown in Fig. 4b. DL-MNs system enabled the quick release of an anti-inflammatory drug (diclofenac) from the fast-dissolving HA layer, followed by a sustained release of an anti-angiogenic agent (DC101) from the crosslinked MeHA layer. The combination of these two drugs ensured both immediate and prolonged therapeutic effects, effectively reducing neovascularization by over 90% compared to single-phase treatments. This dual drug delivery method demonstrates HA’s capability to support complex therapeutic regimens with multiple release phases, providing enhanced efficacy in treating ocular diseases.

One of the challenges with using HA is its relatively low mechanical strength, which can limit its ability to effectively penetrate tougher ocular tissues without bending or breaking. To overcome this, HA is often combined with stronger polymers such as PLA or PLGA, which provide additional structural support while still maintaining the biocompatibility and degradability required for ocular applications. Shi et al. developed HA-PLA MNs to treat fungal keratitis with different PLA contents of 0%, 10%, and 30%^51^. The MNs were fabricated using a micromolding technique. The mechanical strength and dissolution time could be precisely controlled by altering the contents of PLA. The in vitro dissolution of 30% MNs was found to be notably delayed in deformation when comparing 10% content PLA MNs, as shown in Fig. 4c, and sustained release of drug over 8 hours was confirmed. MNs with a 30% PLA MNs showed twice increased insertion force at 200 μm penetration depth comparing with the 10% PLA MNs. PLA contents facilitate effective penetration into the corneal epithelium without causing irritation, and the cornea fully recovered within 12 hours as shown in Fig. 4d.

In conclusion, HA proves to be an exceptional material for ocular drug delivery systems due to its natural biocompatibility, biodegradability, and hydrophilic properties. Its ability to retain moisture and provide minimal irritation makes it ideal for sensitive tissues like the cornea, offering enhanced bioavailability and reducing friction during MN insertion. Furthermore, HA’s versatility allows it to be crosslinked or chemically modified to create hydrogels with controlled and prolonged drug release profiles, which can be fine-tuned by adjusting the molecular weight or crosslinking density. The development of DL-MN systems that leverage the distinct release characteristics of HA and MeHA highlights its potential to deliver complex therapeutic regimens with both immediate and sustained release phases. However, HA’s relatively low mechanical strength remains a limitation, which has been addressed by combining it with stronger polymers such as PLA, enhancing the structural integrity of MNs without compromising biocompatibility. Overall, HA’s adaptability makes it a valuable asset in the development of advanced ocular drug delivery systems, capable of addressing a wide range of therapeutic needs.

### Other Materials MNs

In addition to the materials introduced in previous parts, a variety of other materials have been explored for their unique properties in ocular drug delivery systems, each offering distinct advantages tailored to specific therapeutic needs. The choice of material is often guided by the specific therapeutic demands of the condition being treated—whether it is sustained drug release, enhanced biocompatibility, or immediate dissolution for rapid drug delivery. This diversity allows for a more targeted and effective treatment strategy for a wide range of ocular diseases.

For instance, PVA is widely used in ocular drug delivery due to its unique combination of biodegradability, mechanical strength, and biocompatibility. Compared to synthetic biodegradable polymers like PLA and PLGA, which are often used for sustained-release applications, PVA stands out for its ability to dissolve more rapidly in aqueous environments, making it suitable for short-term or immediate drug release. In this regard, PVA shares a similar fast-dissolving characteristic with HA, which also dissolves quickly upon contact with ocular tissue, allowing for minimal residue and faster healing. PVA can also be used as a holder that connects MNs to a supporting patch, and it dissolves quickly, leaving the penetrated MNs in the tissue^52^.

PVP is another material widely used in MN systems due to its excellent water solubility and ability to form rapidly dissolving structures. MNs proposed by Thakur et al. using PVP showed the dissolving time varied from 60 to 180 seconds by altering the molecular weight of PVP, while both MNs effectively penetrated the corneal and scleral tissues^53^. These MNs were particularly useful in delivering macromolecules, such as fluorescein isothiocyanate-labeled dextrans, which showed significantly enhanced permeation when compared to traditional delivery methods. The study also highlighted the mechanical integrity of high-MW PVP, which provided sufficient strength to penetrate ocular tissues without breaking; however, to achieve MNs capable of penetrating with PVP alone, the use of high-MW PVP is necessary^53,54^, as lower molecular weights do not offer the same mechanical robustness.

In addition, the combination of PVA and PVP in MN systems offers further benefits. PVP’s water solubility makes it ideal for rapid drug release. In combination with PVA’s mechanical strength, it creates a synergistic effect, allowing faster dissolution while maintaining the necessary mechanical properties for effective penetration^55–57^. Albadr et al. showed the PVP combined with PVA MNs showed the twice faster dissolution rate in 30 second insertion test period than PVA only MNs^55^. PVA’s mechanical properties are enhanced, when combined with PVP, allowing for greater flexibility in MN design. This combination provides the MNs with sufficient mechanical integrity to penetrate the corneal surface, ensuring efficient drug delivery without causing significant damage to delicate ocular tissues^56^. The combination of PVA and PVP leverages the mechanical strength of PVA and the rapid dissolving properties of PVP^55^. This partnership ensures that the MNs are strong enough to penetrate tissues effectively while also dissolving quickly once inside the body, ensuring a fast and efficient drug release. These properties complement each other well, particularly in ocular drug delivery, where MNs need to perform reliably without leaving residual material in the eye. Compared to synthetic biodegradable polymers like PLA or PLGA, which are more suited for sustained-release applications, the PVA-PVP combination excels in scenarios where a short-term, high-impact drug release is needed.

Collagen is inherently biocompatible, promoting tissue regeneration and wound healing, which is crucial for treating inflammatory eye conditions such as bacterial keratitis. However, native collagen lacks the mechanical strength required for MN fabrication. The methacrylate modification improves collagen’s ability to form strong, cross-linked structures, ensuring the MNs are sufficiently robust for corneal penetration while maintaining their biodegradability and biocompatibility. Kong et al. proposed MNs with the materials for the treatment of bacterial keratitis^58^. This collagen-based material, known for its excellent biocompatibility, not only facilitated the delivery of antibacterial agents but also promoted tissue regeneration and wound healing in the cornea, making it particularly beneficial for inflammatory conditions.

Poloxamers are thermoresponsive block copolymers that show finely tunable sol-gel transition temperatures^21^. For example, poloxamers remain fluid at room temperature but solidify into gels upon exposure to body temperature, forming in situ implants. This thermoresponsive behavior was particularly advantageous for treating posterior eye conditions, where maintaining consistent and prolonged therapeutic levels is essential for effective treatment. Thakur et al. demonstrated the use of hollow MNs to deliver a poloxamer-based formulation directly into the sclera for the treatment of posterior segment diseases^59^. This method allowed for sustained drug release over 24 h while minimizing invasiveness, making it an attractive option for chronic conditions like macular degeneration or diabetic retinopathy. Characteristics of poloxamers to transition from liquid to gel ensures the drug remains localized at the target site, further improving therapeutic efficacy.

SU-8 is an epoxy-based photoresist known for its high mechanical strength, chemical stability, and excellent biocompatibility, making it a preferred material for microfabrication processes. SU-8 was specifically chosen for the fabrication of MNs due to its rigidity, which was essential for penetrating the thicker layers of the cornea while minimizing impact on surrounding tissues^60^. To further enhance precision and control, the researchers employed a spring-loaded pen-type insertion system, ensuring consistent and localized drug delivery. The rigidity provided by SU-8 allowed the MNs to maintain their shape during insertion, enabling the precise delivery of the therapeutic agent to targeted areas without causing unnecessary trauma to adjacent tissues. This design ensured that the localized drug release was both effective and minimally invasive, optimizing the treatment for conditions like corneal neovascularization.

These varied materials—collagen, poloxamer, PVA, PVP, chitosan, and SU-8—collectively illustrate the wide range of strategies that can be employed in MN-based ocular drug delivery. Each material brings unique characteristics suited to specific clinical needs, whether for rapid dissolution, sustained release, or enhanced mechanical strength. Together, they demonstrate the versatility and growing potential of MNs in addressing the challenges of treating various ocular conditions.

## Fabrication Methods

MNs for ocular drug delivery are produced using a variety of fabrication methods that offer precise control over geometry, drug loading, and release profiles^61,62^. In this section, we specifically focus on wet fabrication techniques, which dissolve polymers in a solvent to shape the MNs and incorporate the drug^6^. These techniques stand out for their ability to fine-tune MN properties, such as mechanical strength and drug release rates, while maintaining biocompatibility^63^. Wet fabrication also facilitates the use of various polymers, enhancing drug encapsulation and sustained release capabilities. This section will explore key methods, including how polymer concentration, solvent evaporation, and layering affect MN performance, highlighting their unique attributes and influence on MN performance in ocular drug delivery.

### Micromolding

Micromolding is one of the most commonly used and versatile methods for fabricating biodegradable MNs. This technique involves creating MNs by casting a polymer solution or melt into pre-fabricated molds that contain the desired MN shape and dimensions. The polymer solution is usually composed of biodegradable materials, such as PLA, PLGA, or natural polymers like HA, which are chosen based on their biocompatibility and controlled degradation properties^64^.

**Fig. 5:**
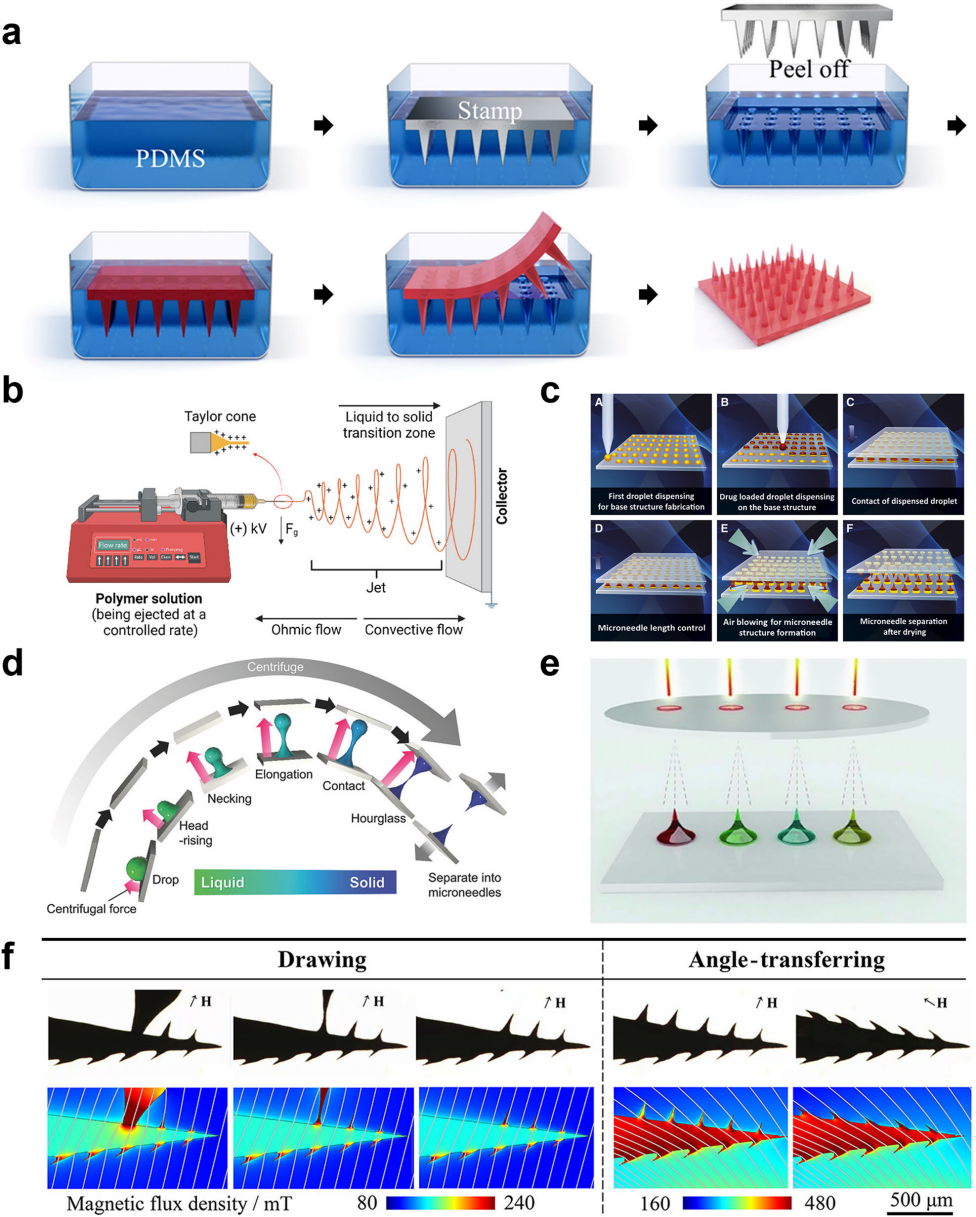
Fabrication methods of MNs for ocular drug deliver. **a** Micromolding. Reproduced with permission from ref. ^69^. **b** Electrospinning. Reproduced with permission from ref. ^78^. **c** Drop-born air blowing. Reproduced with permission from ref. ^89^. **d** Centrifugal lithography. Reproduced with permission from ref. ^95^. **e** Electrolithography. Reproduced with permission from ref. ^100^. **f** magnetorheological lithography. Reproduced with permission from ref. ^102^.

The micromolding process typically consists of several steps. First, a negative mold of the MNs is created, often using materials such as polydimethylsiloxane (PDMS). This mold is produced by lithographic techniques or by directly engraving the MN pattern onto a substrate^65^. Once the mold is prepared, a polymer solution containing the therapeutic agent is poured into the mold cavities. The solution is then allowed to settle, often with the help of vacuum or centrifugal forces to ensure uniform filling of the mold and prevent air bubbles. After filling, the solvent is allowed to evaporate, or the polymer is solidified using techniques like heating or UV curing, depending on the material used. This polymer solidification process is critical because it directly affects the mechanical properties of MNs. In heating-based solidification, the temperature and duration of heating depend on the polymer’s glass transition temperature and thermal stability. For example, biodegradable polymers with solvent, such as PLA or PLGA, are often heated to temperatures around 50–80 °C for several hours to solidify without degrading the drug payload. Meanwhile, UV curing uses ultraviolet light to rapidly harden photosensitive polymers. This method is ideal for polymers such as PVA when combined with a photoinitiator, allowing for fast solidification at room temperature. UV exposure typically ranges from a few seconds to minutes, with the intensity and wavelength of UV light optimized for complete curing without compromising the polymer’s mechanical properties^66,67^. Once the MNs have hardened, they are carefully removed from the mold. In many cases, additional backing layers or adhesive patches may be applied to create a complete MN patch for application. (Fig. 5(a)).

Micromolding is particularly advantageous because it allows for high precision in controlling the shape, height, and sharpness of the MNs^68,69^, which is critical for effective penetration of ocular tissues. Additionally, it offers flexibility in terms of drug loading, as therapeutic compounds can be incorporated into the MN matrix or coated onto the surface^70–72^. This method is scalable and reproducible, making it ideal for producing MNs in large quantities while maintaining consistent quality. The simplicity of micromolding, combined with its ability to produce complex structures, makes it a preferred method for fabricating biodegradable MNs for drug delivery to sensitive areas like the eye^18,52^. A major challenge with micromolding is ensuring precise control over drug distribution within the MNs, as the drug can migrate toward the base during the casting process, which may result in uneven dosing. This can result in inconsistent drug release profiles. For long-term ocular treatments, this uneven distribution can significantly impact therapeutic outcomes. In diseases like glaucoma or retinal disorders, where sustained drug delivery is crucial, variability in drug release can lead to suboptimal treatment. For example, if the drug is concentrated at the base of MNs, it might be released too quickly, resulting in an initial burst release but insufficient sustained delivery over time. This can cause a rapid decline in therapeutic drug levels, requiring more frequent treatments or potentially reducing the efficacy of the therapy^73^. In ocular applications, uneven drug release can also affect safety. If certain areas of MNs release too much drug at once, it could lead to localized tissue toxicity, particularly in sensitive ocular tissues such as the cornea or retina^74^. This makes it especially important for micromolding processes to be optimized for precise and uniform drug distribution, ensuring that the MNs deliver a consistent therapeutic dose throughout the treatment period.

To address these challenges, alternative techniques, such as spray deposition, have been explored. The spray deposition process, particularly the dual-nozzle spray technique^75^, offers unique advantages over conventional drop-casting of solutions into molds. One key benefit is its ability to maintain the structural integrity of sensitive biomolecules, such as proteins, by minimizing exposure to harsh solvents and high temperatures during fabrication. This method not only improves the uniformity of the drug distribution within the MNs but also enhances scalability and precision, making it a promising option for creating MNs with customizable drug release profiles^76,77^.

### Electrospinning

Electrospinning is a versatile and widely used technique for fabricating MNs and other nanostructures due to its ability to produce fine, fibrous materials with high surface area^78–80^. In this process, a polymer solution is charged and ejected through a small nozzle under high voltage, creating a jet that solidifies into fibers as the solvent evaporates. The fibers are then collected on a grounded surface, forming a continuous, porous nanofibrous mat. This method is particularly useful for producing MNs with high drug-loading capacities and controlled release profiles, as the fine fibers can encapsulate drugs within the polymer matrix. Additionally, electrospinning allows for the use of various biodegradable materials, such as chitosan, PLGA and PVA, making it an ideal technique for applications where both structural integrity and biocompatibility are required, such as in ocular drug delivery^81,82^. (Fig. 5(b)).

One of the key advantages of electrospinning is its capacity for incorporating drugs or bioactive molecules uniformly within the fibers, which allows for controlled and sustained drug release^83^. For example, electrospun nanofiber membranes coupled with dissolvable MNs were used to create a Janus-type antimicrobial dressing. This dressing exhibited enhanced efficacy in delivering antimicrobial peptides to biofilms, successfully treating infections in wound models. The electrospinning process allowed for the precise encapsulation of the peptides in the nanofibers, while the MNs provided direct penetration into biofilms, enabling the dual-action system to be highly effective in eradicating bacterial infections^84^. To further expand on the versatility of electrospinning, a novel approach involving the creation of spindles-on-a-string (SOS) hybrid structures has demonstrated significant advancements in drug delivery. This method, developed through a modified coaxial electrospinning process, enables a biphasic drug release system. By integrating PEG spindles with ethyl cellulose nanofibers, the structure facilitates an initial burst release of the drug followed by a sustained release. This biphasic release profile enhances therapeutic efficacy by providing both immediate and prolonged delivery of poorly water-soluble drugs, such as ibuprofen. The SOS hybrid system showcases the potential of electrospinning to create complex, functional nanofiber structures that can optimize drug release and address specific therapeutic needs^85^.

Moreover, the advancement of fiber-reinforced silk fibroin MN technology has achieved notable improvements by integrating specialized fibrous materials into the MNs, significantly boosting their mechanical durability and ability to adhere to tissues. Wang et al., have developed a novel fiber-reinforced silk MN patch designed to improve tissue adhesion for the treatment of diabetic wound infections. The innovation lies in the combination of chitosan fibers and silk fibroin, which enhances the mechanical properties and adhesive strength of the MNs, addressing the common issue of poor mechanical integrity in hydrogel MNs. Additionally, the patch offers a synergistic antibacterial effect, enhanced by the incorporation of epigallocatechin gallate, which further promotes wound healing by regulating macrophage polarization and accelerating tissue repair^86^. As this technology advances, it shows significant potential for a range of medical applications, such as precision drug administration, improved wound healing, and addressing localized infections^87,88^.

Despite its advantages, electrospinning has several limitations when applied to MN fabrication. One of the primary challenges is the difficulty in precisely controlling fiber alignment, which can lead to inconsistent mechanical properties in the final product. Additionally, the process is highly sensitive to environmental conditions, such as humidity and temperature, which can affect fiber morphology and overall production consistency. The scalability of electrospinning is another concern, as it can be challenging to produce large quantities of MNs with uniform characteristics. Furthermore, while electrospinning allows for high drug loading, achieving controlled and sustained release over extended periods can be difficult, especially when targeting specific therapeutic applications like ocular drug delivery.

### Droplet-born Air Blowing

The droplet-born air blowing (DAB) method is a unique technique for fabricating dissolvable MNs, where polymer droplets are shaped into MNs using controlled air blowing. This approach allows for precise control of MN size and drug dosing by adjusting the droplet volume and polymer concentration. The method’s gentle processing conditions, which occur at room temperature without the use of heat or UV light, make it ideal for fabricating MNs with sensitive biological drugs, such as proteins or peptides, without compromising their activity. Additionally, the process is quick, with fabrication times under 10 minutes, offering an efficient and scalable solution for drug delivery applications. (Fig. 5(c)).

This technique is especially effective for producing MNs from high-viscosity polysaccharide solutions. In a study utilizing the DAB method, Kim et al., successfully fabricated insulin-loaded dissolvable MNs designed for diabetes treatment. The process involved dispensing a polymer solution containing insulin onto a flat surface, followed by the application of air blowing to shape the droplet into a MN. This approach resulted in a two-layered MN structure that ensured the complete delivery of insulin into the skin without any drug loss. The study demonstrated the bioavailability and efficacy of the insulin-loaded MNs, showing similar glucose-lowering effects to subcutaneous insulin injections in diabetic mice. The DAB method also preserved the stability of insulin during fabrication and storage, confirming the method’s suitability for delivering biologics^89^.

In this sense, the DAB method, which shapes MNs at room temperature, is ideal for preserving the stability of temperature-sensitive ocular drugs. In treating corneal neovascularization, maintaining the integrity of proteins that inhibit abnormal blood vessel growth is crucial. This gentle process helps maintain the bioactivity of therapeutic agents, such as anti-VEGF proteins used in treating corneal neovascularization, ensuring sustained, localized drug release without degradation during fabrication^90^. Likewise, for retinal diseases, such as age-related macular degeneration, proteins like monoclonal antibodies must remain stable to ensure long-term, localized therapeutic effects^91^. The use of controlled, mild fabrication techniques in MNs production could allow these drugs to be encapsulated and delivered directly to the eye with minimal loss of bioactivity, ensuring effective, sustained treatment while reducing the need for repeated invasive procedures.

The DAB method’s ability to preserve drug bioactivity stems from its gentle and streamlined process. By eliminating harsh solvents and operating at room temperature, it minimizes risks such as denaturation, aggregation, or degradation of proteins and peptides. Additionally, the precise control over droplet size and polymer concentration allows for uniform encapsulation of sensitive molecules. The air-blowing technique further ensures that the drug remains embedded within the polymer matrix in a manner that reduces direct exposure to external stresses, maintaining molecular stability. These advantages are particularly promising for ocular applications, where therapeutic agents must retain their functionality to provide effective treatment over extended periods. While specific studies on ocular biologics like anti-VEGF proteins using the DAB method remain limited, its demonstrated potential in encapsulating sensitive molecules suggests that this technique could ensure consistent bioavailability and therapeutic outcomes in such applications.

Furthermore, a novel approach combining electrospinning with the DAB technique has been developed to enhance the performance of dissolvable MNs. By fabricating MNs on an electrospun fibrous sheet supported by a pillar array, this method improves the mechanical strength and insertion efficiency of the MNs. The electrospun fibers provide a porous structure that not only supports the MNs but also allows for rapid separation upon skin insertion. This integrated system significantly reduces the time required for MN implantation and drug release, making it highly suitable for applications requiring fast and efficient transdermal delivery^92^.

Despite its advantages, the DAB method has some limitations. One challenge is achieving uniform MN shapes, especially when scaling up the process for mass production. Additionally, while the air-blowing technique ensures rapid solidification, it may result in variations in MN mechanical properties, potentially affecting skin penetration and drug delivery consistency. Finally, the need for specialized equipment for precise air-blowing control adds complexity to the manufacturing process, which could impact its wider adoption. To improve the DAB method, automated droplet dispensing systems would enhance the uniformity of MN shapes by ensuring consistent droplet size and placement. Automated air-blowing systems, with precise control over air pressure and duration, can further improve the mechanical properties and structural consistency of the MNs^93^.

### Centrifugal Lithography

Centrifugal lithography (CL) is an advanced MN fabrication technique that utilizes centrifugal force to mold polymers into precise MN shapes. One of the key advantages of this method is its ability to produce high-resolution, uniform MNs with fine control over size and geometry. The centrifugal force ensures even distribution of the polymer solution within the mold, leading to consistent MN structures. Additionally, this technique is highly scalable, making it suitable for mass production. It also allows the use of various biodegradable polymers, which can be easily integrated with drugs or bioactive compounds for controlled release, making it particularly useful for biomedical applications like ocular or transdermal drug delivery^94^. (Fig. 5(d)).

Yang et al. presented the use of CL to fabricate dissolvable MNs encapsulating biopharmaceutics, such as tuberculin purified protein derivatives (PPD), in a one-step process. The technique leverages centrifugal force to shape polymer drops into MNs without the need for additional tools or external stimuli like UV light or heat, preserving the activity of sensitive biological materials. The PPD-loaded MNs were successfully used in a tuberculin skin test, showing comparable immune responses with less skin damage compared to conventional injections^95^. Building on the previous research from the same group using CL, Lahiji et al. introduced a novel tissue interlocking dissolving MN (TI-DMN) design to further enhance the precision and efficiency of transdermal drug delivery. The TI-DMN, featuring a unique narrow-neck and wide-body geometry, allows the MNs to interlock with skin tissues more effectively, reducing the risk of dislodgment in dynamic tissues. This innovation improves upon the earlier work by ensuring stronger adhesion to the skin and enhancing drug delivery efficiency. The interlocking design significantly reduced MN displacement under conditions such as bending or stretching, which conventional MNs struggled to maintain^96^. In a comparative study between CL and DAB, CL demonstrated clear advantages in preserving the activity of encapsulated drugs. Both methods resulted in MNs with similar physical properties and fracture forces, but CL proved more effective at maintaining the stability of fragile biological compounds. For example, after 24 hours, CL retained 75.32% of epidermal growth factor activity, whereas DAB retained only 41.75%^97,98^. The use of lower temperatures and vacuum drying in CL minimizes stress on the drugs, making it particularly suitable for applications that require the preservation of therapeutic efficacy.

CL would offer unique advantages that make it particularly promising for ocular drug delivery, especially when compared to other MN fabrication techniques. Its ability to use centrifugal force for self-shaping MNs ensures precise, symmetrical structures that can be fine-tuned to achieve the optimal geometry for the curved and sensitive surface of the eye. Furthermore, CL can easily produce MNs with a wide base that gradually narrows to a fine tip, allowing for better stability and controlled insertion into ocular tissues. This precision, combined with the ability to encapsulate fragile biopharmaceuticals in a single step, preserves the therapeutic efficacy needed for delicate ocular environments. Furthermore, the centrifugal solidification process avoids harsh conditions like heat or UV exposure, which is particularly important when working with heat-sensitive ocular drugs. These distinct features make CL a strong candidate for advancing minimally invasive, long-acting drug delivery to the eye.

### Electro- and Magnetorheological Lithography

Electrolithography is a cutting-edge MN fabrication technique that utilizes electrohydrodynamic forces to directly shape polymer droplets into MNs without the need for molds. The process involves applying a controlled electric field to a polymer drop on a flexible substrate, which stretches the droplet into a MN shape as shown in Fig. 5(e). This method also allows for precise control over MN geometry by adjusting parameters such as droplet volume and distance from the electric field, resulting in MNs with high aspect ratios and sharp tips. Furthermore, electro-drawing lithography is highly versatile, enabling the encapsulation of both hydrophobic and hydrophilic drugs within the MN structure^99^. This property is achieved by forming porous microstructures, which can be tailored to control drug release profiles^100^. Ruggiero et al. developed a novel setup for electrolithographic MNs with controlled shape and dimensions, addressing the limitations of previous methods such as pedestal formation and lack of parallel processing. The introduction of titanium micro-heaters integrated into the pyroelectric substrate allowed for uniform electric field generation, enabling parallel fabrication of MNs without the formation of a pedestal at the base. Additionally, the process was optimized through a double-step electro-drawing protocol to prevent MN shrinkage while maintaining precise control over their shape and size, making the method suitable for scalable production^101^.

Magnetorheological lithography (ML) is a novel MN fabrication technique that leverages the properties of magnetorheological fluids which are smart materials that change their viscosity and flow characteristics in response to magnetic fields as depicted in Fig. 5(f). The basic principle involves using an external magnetic field to control the alignment and structuring of magnetic particles within a polymer matrix. This allows for the precise molding of MNs with customizable shapes and sizes, depending on the strength and orientation of the applied magnetic field. Once the MNs are shaped, the polymer is solidified, often through UV curing or heat treatment, locking the magnetic particle-aligned structure in place. By manipulating the magnetic field, the alignment of particles within the matrix can be controlled, leading to improvements in MN strength, durability, and precise geometry. This method also enables the production of MNs with complex internal structures, which can be used to create channels or reservoirs for controlled drug delivery. In addition to its precision, ML is highly versatile, accommodating a wide range of polymeric materials and magnetic fillers. This flexibility allows for the integration of biodegradable and biocompatible materials, making it ideal for biomedical applications such as transdermal, ocular drug delivery. Chen et al. fabricated a bioinspired MN fabrication technique using ML to mimic the honeybee stinger’s microbarbed structure. By applying an external magnetic field, MNs with tilted microbarbs were directly drawn, offering easy skin insertion but challenging removal due to tissue adhesion. The study demonstrated that these barbed MNs required significantly less force for insertion but much more force for removal compared to barbless MNs, making them ideal for applications requiring tissue adhesion, such as drug delivery and biosignal recording^102^. The microbarbs allow the MNs to interlock with tissues, which is particularly beneficial in dynamic environments like the eye. This ensures that the MNs remain securely in place despite eye movements, reducing the risk of dislodgement during drug administration. Given these characteristics, the microbarbed MNs show great potential for ocular drug delivery applications, providing stable and sustained drug release directly into the target tissue^103^.

In summary, the various fabrication methods for MNs provide several key advantages for ocular drug delivery. These techniques offer precise control over MN geometry and drug release profiles, ensuring effective drug administration. These methods also support scalability, biocompatibility, and the ability to incorporate various therapeutic agents. Furthermore, innovations like rapid drug release or sustained delivery make these methods adaptable for a wide range of ocular applications, enhancing therapeutic outcomes. However, ensuring MN stability during and after fabrication is equally critical, as environmental factors such as humidity and temperature can compromise their structural integrity or drug efficacy. Additionally, microbial biofilm formation during storage poses a risk of contamination, potentially reducing therapeutic effectiveness. To address these challenges, strategies such as maintaining controlled fabrication environments, using sterile handling procedures, and incorporating antimicrobial agents into MN materials are being actively explored to preserve both stability and sterility, ensuring safe and reliable drug delivery^104–106^. A summary of the fabrication methods discussed in this section, including the corresponding MN materials, dimensions, mechanical strength, drug loading capacity, and release profiles, is provided in Table 1.

**Table. 1:** Summary table containing the information discussed in fabrication methods.

Fabrication method	Material	Size (Base diameter, height) (µm)	Mechanical strength (N)	Drug/protein loading capacity (µg)	Drug releasing period	Ref
**Micromolding**	PLGA	300, 600	~ 0.3	20 ~ 80	~ 50 days	^72^
	HA	250, 500	~ 0.4	2	~ 120 h	^18^
	PLA, HA	200, 390	~ 300	-	~ 8 h	^51^
**Droplet-born air blowing**	CMC, HA, PVP	300, 200 ~ 600	~ 0.5	50	~ 2 h	^89^
	CMC	190, 445	~ 0.36	10	-	^98^
**Centrifugal lithography**	CMC	140, 490	~ 0.24	10	-	^98^
**Electrolithography**	PLGA	500, 400 ~ 800	-	-	-	^100^
	PLGA, PVP, VA	300, 300	~ 0.12	-	~ 3 h	^99^
**Magnetorheological Lithography**	Curable liquid with iron particle	350, 300 ~ 700	~ 0.2	-	-	^103^

## Drug Release Mechanisms for Ocular Applications

In MNs designed for ocular applications, various release mechanisms, such as diffusion, dissolution, degradation, swelling, and stimulus-triggered release, play a crucial role in regulating drug delivery. The effectiveness of these mechanisms is governed by factors such as material composition, drug encapsulation method, and the interaction between MNs and ocular tissues. For instance, polymer crystallinity, molecular weight, and cross-linking density can significantly influence degradation rates and drug release profiles. Similarly, the physicochemical properties of the drug, such as solubility and molecular size, determine its release kinetics. These mechanisms are deeply influenced by the interplay between the material properties of MNs and the characteristics of the drug, allowing precise control over both the rate and duration of release. For example, biodegradable polymers like PLGA and hyaluronic acid facilitate controlled degradation and safe absorption, while hydrogels enable swelling-based systems for sustained delivery. Stimulus-responsive materials, on the other hand, allow for on-demand release triggered by external factors such as pH, light, or temperature. Each mechanism is influenced by the material properties of the MNs and the characteristics of the drug being delivered, allowing for tailored solutions to meet specific therapeutic needs. To achieve effective drug release, advanced strategies such as nanoparticle incorporation, multilayer designs, or stimulus-triggered formulations are often employed, enabling precise temporal and spatial control over drug delivery. These approaches are particularly critical in addressing challenges such as premature drug release or insufficient bioavailability. Advanced drug delivery systems aim to control not only the rate of drug release but also its spatial distribution, ensuring that the therapeutic agent reaches the target area efficiently and with minimal side effects. These innovations not only enhance therapeutic precision but also minimize risks such as inflammatory responses or local toxicity, which are critical considerations in ocular applications. This precision is vital for achieving optimal therapeutic outcomes in the treatment of ocular diseases, where both the method of release and the material used can have a significant impact on the effectiveness of the treatment. In the following sections, we will briefly explore the key drug release mechanisms employed in ocular MNs.

**Fig. 6:**
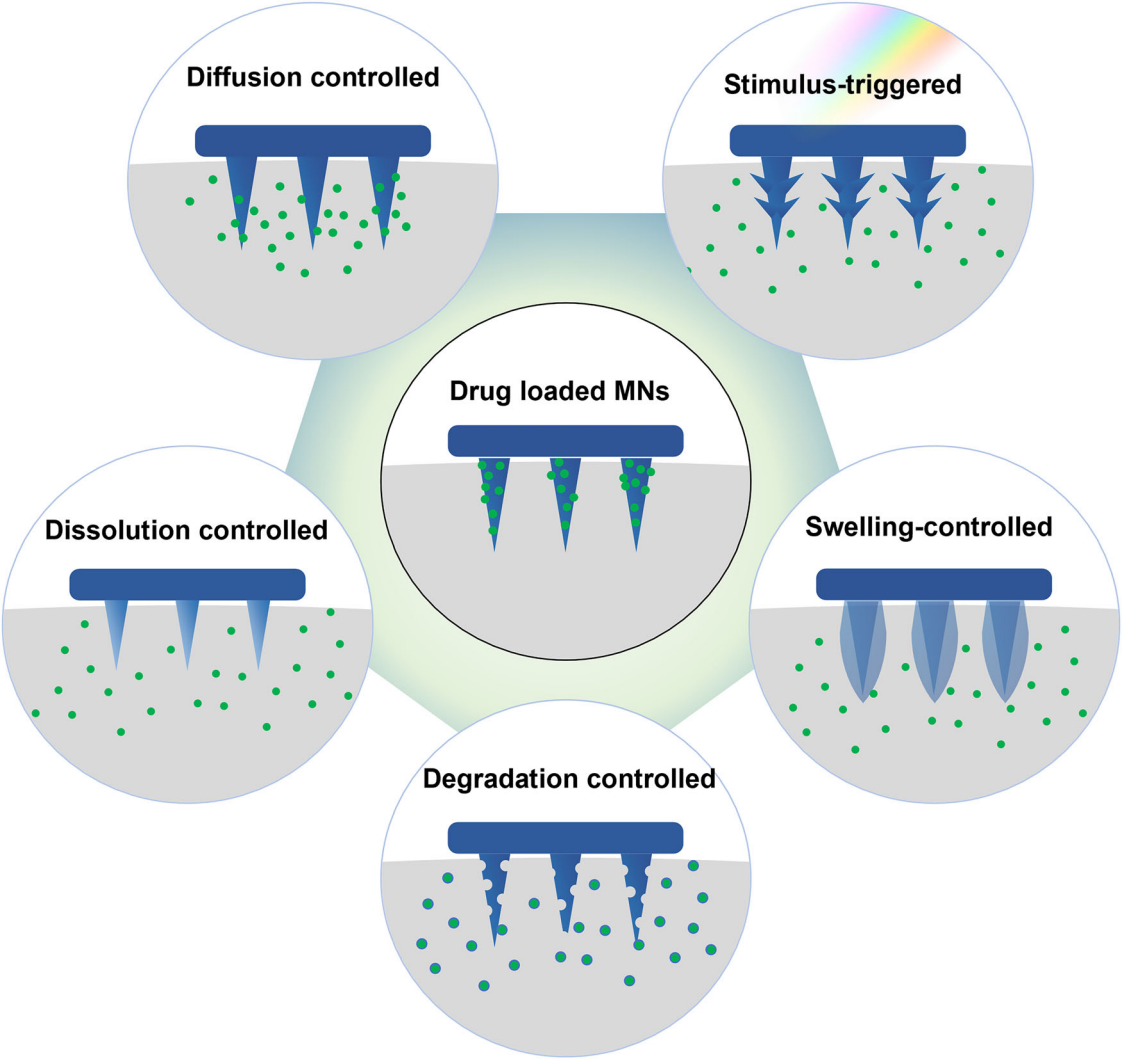
Representative drug release mechanisms for ocular drug delivery MNs; diffusion-controlled release, dissolution-controlled release, degradation-controlled release, swelling-controlled release, stimulus-responsive release.

Figure 6 illustrates the five primary drug release mechanisms employed in microneedle (MN)-based ocular drug delivery systems. Each mechanism offers distinct advantages depending on the therapeutic requirements and material properties. Diffusion-controlled release relies on the gradual movement of the drug through a porous matrix or coating, making it ideal for steady, long-term delivery but limited by its dependency on material porosity and drug solubility. Dissolution-controlled systems utilize rapidly dissolving materials like PVP, providing an immediate and fast release profile, suitable for acute treatments but less effective for sustained therapies. Degradation-controlled release employs biodegradable materials such as PLGA, offering a balance between sustained delivery and biocompatibility; however, the degradation rate can be influenced by environmental factors, leading to variability in drug release. Swelling-controlled systems, often based on hydrogel matrices, enable a gradual drug release as the material absorbs fluid and expands, offering mechanical flexibility but potentially slower onset times. Lastly, stimulus-triggered systems, using materials responsive to external triggers such as pH or temperature, enable precise and on-demand drug delivery but require more complex designs and external control mechanisms. While diffusion, dissolution, and degradation-based systems are generally simpler and more predictable, swelling and stimulus-triggered mechanisms provide greater control and adaptability for advanced applications. This comparative analysis highlights how each mechanism can be optimized for specific clinical needs, emphasizing the versatility of MN technology in ocular drug delivery.

### Diffusion-Controlled Release

Diffusion-controlled drug release occurs when drug molecules move from an area of high concentration (within the delivery system) to a lower concentration in the surrounding tissues as shown in Fig. 6. This movement is driven by the natural tendency of molecules to equalize concentration gradients and is primarily governed by Fick’s laws of diffusion^107^. Porous and coated MNs are representative examples for explaining diffusion-controlled drug release.

In porous MNs, the drug is stored within the porous structure of the MN itself. Once the MN is inserted into the tissue, interstitial fluid enters the pores, dissolves the drug, and allows it to diffuse through the interconnected channels within the MN^15^. The release rate in PMNs depends heavily on factors such as pore size, porosity, and the chemical properties of the material used to form the MN. Materials including Si and metals have been employed to fabricate PMNs, each offering different levels of biocompatibility and release control^34,108^. While porous structures are effective for sustained release, they may reduce the mechanical strength of the MN, potentially posing risks such as breakage upon insertion^109^. Coated-MNs (CMNs), on the other hand, rely on applying a drug coating to the surface of solid MNs. The drug release is primarily dependent on the physicochemical properties of the coating layer and its interaction with the MN substrate^15^. The drug release profile can be controlled by adjusting factors such as the thickness of the coating or the inclusion of excipients that regulate the dissolution rate^110^.

In both PMNs and CMNs, diffusion-controlled release offers a promising mechanism for sustained, localized drug delivery. By adjusting structural elements in PMNs or modifying coating properties in CMNs, the rate and profile of drug release can be finely tuned to meet therapeutic needs. These MN systems provide versatility in delivering drugs over extended periods, making them highly valuable in various medical applications.

### Dissolution-Controlled Release

Dissolution-controlled release refers to the mechanism in which a drug is embedded within a carrier material that dissolves when exposed to biological fluids as shown in Fig. 6. In the case of MNs, this occurs when the needle matrix dissolves upon insertion into the skin or other tissues, releasing the drug in a controlled manner. The release rate can be adjusted by selecting appropriate matrix materials and controlling the distribution of the drug within the MN structure.

A prime example of this is Dissolving MNs, which dissolve upon contact with interstitial fluid. The release kinetics of the drug can be fine-tuned by adjusting the composition of the MN matrix or by controlling the drug distribution within the MN system itself^111^. For example, MNs made from PVA have been shown to control diffusion rates and deliver drugs over extended periods by altering the molecular weight of the matrix material; higher molecular weight of PVA reduced the solubility of the MNs, resulting in slower drug release^112^. Another notable example is a dissolving MN patch made from PLA and HA developed for the treatment of fungal keratitis. In this system, the PLA-HA MNs dissolve upon contact with the tear fluid, delivering antifungal drugs to the corneal tissue. The dissolution rate of the MNs can be controlled by adjusting the concentration of PLA in the MN matrix. Increasing the PLA content slows down the dissolution rate due to the higher mechanical strength and slower biodegradation of PLA compared to HA^51^.

### Degradation-Controlled Release

Recent advancements in biodegradable materials, including electrochromic devices with eco-friendly degradation mechanisms, self-deployable electronic implants designed to dissolve after use, and highly sensitive strain sensors with fully degradable components for biomedical applications, highlight the growing importance of controlled degradation in medical and electronic systems^113–115^. Similarly, in drug delivery applications, degradation-controlled release plays a pivotal role in ensuring sustained therapeutic effects, eliminating the need for device removal, and minimizing long-term side effects. Degradation-controlled release involves using biodegradable materials that gradually break down when exposed to biological environments as shown in Fig. 6, releasing the encapsulated drug over time. This method eliminates the need for removing the drug delivery system after the release is complete, as the materials degrade into non-toxic byproducts. The degradation process can occur through mechanisms such as hydrolysis, enzymatic activity, or solubilization, depending on the material composition^15^. This mechanism is especially useful for long-term therapies where sustained release over weeks or months is required. The key to this system lies in selecting the appropriate materials and designing the drug formulation to achieve the desired degradation and release profiles.

Naturally degradable materials like silk and chitosan are often used as encapsulating materials to maintain drug stability while providing excellent mechanical properties. For example, silk fibroin MNs can be processed under mild aqueous conditions, and their degradation rate can be adjusted through treatments such as ethanol exposure, which increases the crystalline structure and slows the release rate^15^. For more precise control of drug release profile drug-loaded biodegradable particles are fabricated using materials like PLA and PLGA. For instance, a core-shell MN system with a drug-loaded PVP core and a PLGA shell can provide a preprogrammed burst release over several days to months for transdermal applications, depending on the PLGA composition^72^. Similarly, PVA-PVP bilayer MN loaded with PLGA nanoparticles was developed for sustained drug delivery to the posterior segment of the eye. The incorporation of nanoparticles in this system allowed for controlled and extended drug release over more than two months. The nanoparticles enhanced the drug release profile by shielding the drug from immediate exposure to the biological environment and by providing a sustained release mechanism as the PLGA nanoparticles gradually degraded^116^.

### Swelling-Controlled Release

In swelling-controlled systems, the drug is encapsulated in a polymer matrix that swells upon contact with water or other bodily fluids. This swelling increases the pore size of the matrix, allowing the drug to diffuse out as shown in Fig. 6. This mechanism is most commonly associated with hydrogel MNs, which transition from a glassy to a rubbery state upon contact with interstitial fluid, enabling the controlled release of drugs over time. The release kinetics depend heavily on the swelling ratio of the hydrogels, which is influenced by factors such as crosslinking density and the chemical structure of the polymer matrix^15^.

Hydrogel-embedding MNs consist of hydrophilic, three-dimensional polymer networks that can absorb significant amounts of fluid. These hydrogels, which can be physically or chemically crosslinked, swell after application, allowing the encapsulated drugs to be released gradually^15^. Than et al. proposed a DL-MN system that uses HA for the inner core and MeHA for the outer layer. In this system, HA dissolves quickly, providing an initial burst of drug release, while the MeHA layer swells, controlling the release of the drug over time^18^. The swelling of MeHA slows down the release, creating a sustained effect after the initial rapid dissolution of HA. This design exemplifies a swelling-controlled release system, where the MeHA’s swelling regulates the prolonged release of the drug.

Despite their advantages, swelling-controlled hydrogel MNs face challenges, including maintaining mechanical strength in high-humidity environments and the potential deposition of matrix materials in the eye. However, they offer significant promise in achieving controlled, sustained drug release with the potential for diverse medical applications. Future innovations could focus on developing user-friendly, wearable patches for longer-term use and integrating stimuli-responsive hydrogels for trigger-controlled drug delivery.

### Stimulus-responsive Release

As shown in Fig. 6, Stimulus-responsive release mechanisms are based on external or internal stimuli such as pH, temperature, light, or magnetic fields that trigger the release of the drug^20^. This approach offers highly precise control over drug release, making it especially promising for ocular therapies that require on-demand drug delivery. The stimuli trigger a structural or chemical change in the MN matrix, enabling drug release only when needed, which enhances therapeutic effectiveness and reduces side effects.

One example of endogenous stimuli-responsive MNs involves materials that respond to pH variations. MNs designed with pH-sensitive materials can take advantage of this by releasing drugs specifically when local environment become acidic. For instance, a pH-responsive MN system encapsulated with cisplatin-loaded nanoparticles was developed for cancer treatment. The nanoparticles remained stable under normal physiological conditions but released the drug more rapidly in the acidic tumor environment, improving the anti-tumor efficacy^117^. Additionally, light-responsive MNs allow for precise control over the timing and location of drug release by applying light at specific wavelengths. For example, near-infrared (NIR) light-responsive MNs for drug delivery have been developed for combined chemotherapy and photothermal therapy. In this system, the NIR light triggers the release of chemotherapy drugs while also raising the local temperature to destroy cancer cells, providing a synergistic therapeutic effect^19^. Similarly, a thermal-responsive hollow MN system loaded with poloxamer-based formulations was developed for intrascleral drug delivery. The poloxamer solution, which remains liquid at room temperature, forms an in-situ gel when exposed to physiological temperature (37 °C), enabling sustained drug release within the sclera. This system offers a minimally invasive method for delivering drugs to the posterior segment of the eye and can provide controlled drug release over extended periods^59^. These examples illustrate how stimulus-responsive MN systems can be customized for different medical applications, offering controlled, targeted drug delivery in response to both internal and external triggers.

## Implantation Methods of MNs into Ocular Tissues

In ocular drug delivery, ensuring the precise and effective implantation of biodegradable MNs loaded with therapeutic agents into the targeted area of the eye is crucial for optimal treatment outcomes. Given the sensitive and dynamic nature of ocular tissues, accurate placement of the MNs is essential to ensure controlled drug release and avoid potential damage to surrounding structures. With biodegradable MNs, this precision becomes even more critical, as they gradually dissolve and release their drug payload over time. Unlike non-degradable systems, biodegradable MNs cannot be repositioned or removed once inserted, making misplacement a significant concern that could lead to ineffective treatment or unintended tissue exposure. Furthermore, the degradation rate and drug release profile are closely linked to the local environment, so proper placement is vital to maintaining consistent therapeutic effects while minimizing side effects. This makes the careful selection of implantation methods particularly important for biodegradable systems in ocular applications^14,118,119^. In this section, we will discuss various methods for implanting biodegradable MNs into the eye, including patch-based systems, contact lens-integrated MNs, and the use of detachable pens for direct implantation.

### Patch-based System

**Fig. 7:**
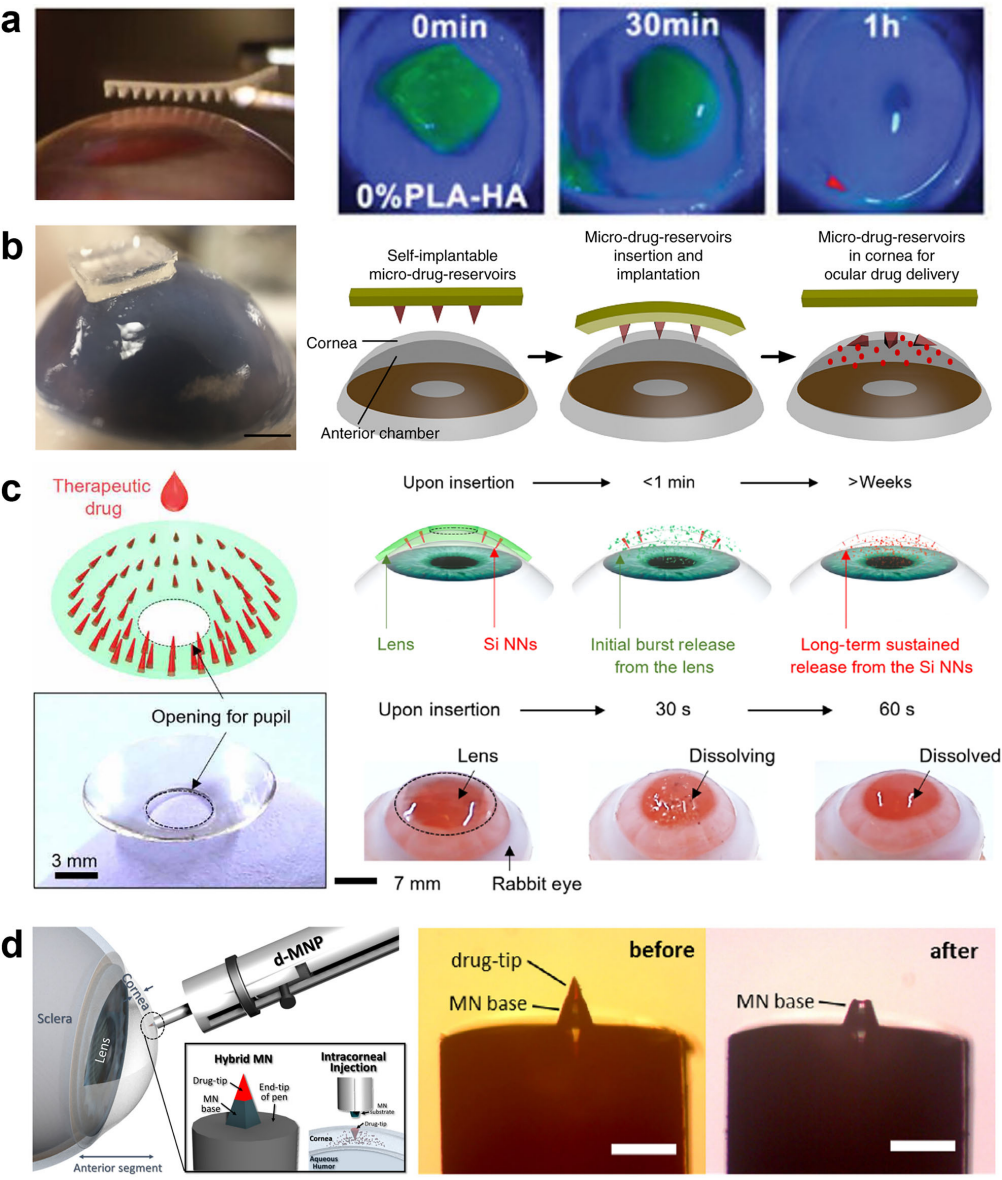
Implantation methods of MNs into ocular tissues. **a** Biodegradable MN patch for corneal healing. Reproduced with permission from ref. ^51^. **b** MNs for ocular drug delivery. Reproduced with permission from ref. ^18^. **c** Contact lens shaped system with silicon nanoneedles for ocular drug delivery. Reproduced with permission from ref. ^36^. **d** Detachable MN pen. Reproduced with permission from ref. ^42^.

Patch-based ocular drug delivery using biodegradable MNs offers a non-invasive and highly controlled method for administering therapeutics directly to the ocular surface. These patches are designed to conform to the surface of the eye, allowing for precise placement of MNs into specific ocular tissues, such as the cornea or sclera. One of the key advantages of this method is its ease of application, as the patch can be applied and removed without the need for specialized equipment or procedures. This method also enables controlled and sustained drug release as the MNs gradually dissolve, ensuring consistent therapeutic dosing over time. Additionally, patch-based systems reduce the risk of infection or injury compared to more invasive techniques, making them a safer and more patient-friendly option for treating a variety of ocular conditions^8,62^. (Fig. 7(a)).

In this respect, biodegradable MNs integrated into patches have emerged as a promising method for ocular drug delivery. These systems are designed to combine the ease of patch application with the precision of MNs to ensure effective drug administration directly to the target tissues. The initial approach involves integrating the MNs directly into a patch, which allows for easy application to the ocular surface. Once applied, the MNs penetrate the corneal or scleral tissue to deliver the drug payload, after which the entire patch can be removed, leaving the MNs embedded in the tissue for drug release. For instance, in a study utilizing a dissolvable MN patch made from HA and PLA, the patch dissolved within 60 seconds of application, making it both fast-acting and efficient. This rapid dissolution minimizes the risk of irritation or patient discomfort while ensuring that the drug remains in contact with the ocular tissue long enough for effective absorption. Additionally, the dissolvable nature of the patch eliminates the need for invasive removal, making it a convenient and patient-friendly option for long-term ocular treatments. This characteristic also allows for more precise control over drug dosing, as the MNs deliver the entire drug load as they dissolve^51^.

Building on this concept, a more advanced technique has been developed where the patch separates from the MNs after insertion, leaving the MNs in place while the back plate is removed. This allows for the MNs to continue delivering drugs without the discomfort of having the patch remain on the eye. Such designs offer greater flexibility and patient comfort, ensuring that the MNs remain securely in the target tissue while the patient’s vision is unimpeded^8^. Alimardani et al. developed a dissolvable MN system where the backplate is designed to dissolve faster than the MNs themselves. The MNs, made from a thermosensitive copolymer, form in-situ nanomicelles upon contact with ocular tissues, allowing for the effective delivery of hydrophobic drugs like dexamethasone. The rapid dissolution of the backplate enables the MNs to remain embedded in the sclera after application, ensuring localized and sustained drug release without the need for the patch to remain in place^120^.

To further enhance this concept, a new generation of patches has been developed that focuses on rapid MN separation from the back plate immediately upon insertion. This ensures that the MNs are left behind in the ocular tissue with minimal discomfort or manipulation, while the patch itself is removed quickly and easily. This rapid separation technology simplifies the application process and ensures more reliable drug delivery. Li et al. introduced a rapidly separable MN patch designed for sustained drug release, utilizing a unique bubble structure between the MNs and the patch backing. The air bubble enables efficient MN penetration under compression, followed by quick detachment under shear force, which allows the MNs to remain embedded in the tissue while the patch is easily removed. This design ensures rapid separation within seconds, enhancing patient comfort and compliance. The MNs, made from biodegradable polymers such as PLGA and PLA, provide a sustained release of the drug over an extended period. This system offers a practical solution for applications requiring long-term drug release with minimal intervention^52,121^.

Typically, these MNs are designed to dissolve quickly, releasing the drug in a short period. However, for conditions requiring sustained drug release, DL-MNs have been introduced. These MNs are composed of two layers: the outer layer dissolves rapidly to provide an initial burst of drug release, while the inner layer dissolves more slowly, allowing for extended drug delivery over time. This approach is ideal for ocular conditions that benefit from prolonged therapeutic effects, ensuring that the drug remains active in the targeted area for an extended period. Than et al. focused on the development of a self-implantable, DL-MN patch for controlled ocular drug delivery. The MNs, designed as micro-drug reservoirs, enable biphasic drug release—an initial burst followed by a sustained release. The outer layer of the MNs dissolves slowly, providing prolonged therapeutic effects, while the inner layer dissolves rapidly to deliver an immediate dose. This system enhances the treatment of conditions like corneal neovascularization, with the study showing a ~90% reduction in neovascular area compared to traditional eye drop methods. The minimally invasive design and ease of application make it suitable for home-based treatments^18^.

### Contact Lens-shaped System

In the development of ocular drug delivery systems, contact lens-shaped biodegradable MNs system presents a novel and promising approach. Contact lens-shaped applications are designed to conform to the curved surface of the eye, offering consistent MN insertion with minimal discomfort while combining the therapeutic advantages of MNs with the convenience and comfort of contact lenses for precise drug delivery directly to the corneal surface^122,123^. By integrating MNs into the structure of a contact lens, the system can ensure controlled and sustained release of drugs while maintaining close contact with the eye. This approach not only improves drug bioavailability but also enhances patient compliance, as the familiar form factor of a contact lens reduces discomfort and simplifies application^124^. Contact lenses offer a unique advantage for treating ocular infections due to their ability to provide prolonged drug contact with the eye’s surface, which enhances drug bioavailability^125^. Unlike eye drops, which are often washed away by tears, contact lenses can maintain sustained drug release and ensure that the medication stays in contact with the cornea for extended periods. For example, amphotericin B was used to treat fungal keratitis, with the MNs made from biodegradable materials such as PVA and PVP. The MNs embedded in the contact lens allowed the drug to be released gradually over time, providing prolonged exposure to the infected corneal tissue. This approach ensured effective treatment by maintaining therapeutic levels of the drug at the site of infection improving drug efficacy^126^.

Moreover, Park et al. demonstrated a significant advancement in the field of ocular drug delivery through the integration of biodegradable Si NNs with a PVA contact lens. The Si NNs are fabricated using microelectromechanical systems (MEMS) techniques on a Si wafer, allowing for precise control over needle geometry. Following this, the NNs are transferred onto the PVA film, transforming the structure from a rigid Si wafer to a flexible, tear-soluble contact lens form. This transition enables the needles to penetrate the corneal surface minimally invasively while the PVA lens dissolves rapidly, leaving the NNs in place for sustained drug release over extended periods^36^. (Fig. 7(b)).

However, contact lens-shaped systems are not without challenges. One major safety concern is the risk of eye irritation caused by prolonged contact between the lens and the ocular surface, especially if the lens edges are not well-designed or if the MNs create localized pressure. Lens opacity can also impair vision during wear, necessitating the use of highly transparent materials. Additionally, improper handling during application or removal could increase the risk of infection, particularly if the lens or MNs are not manufactured and stored under sterile conditions. To mitigate these risks, biocompatible and transparent materials like PVA and hydrogel composites are preferred for lens construction. Sterile fabrication processes, single-use designs, and user-friendly application protocols further reduce infection risks and enhance safety. Design optimizations such as rounded lens edges and tailored MN geometries can minimize mechanical irritation, ensuring patient comfort and compliance.

### Detachable MN Pen

The MN pen system was first introduced to address the challenge of delivering drugs effectively to small and curved ocular tissues, such as the cornea, where conventional MN patches and applicators often fail due to irregular surfaces and lack of support. The pen employs a spring-loaded applicator designed to ensure consistent, impact-based insertion of MNs, overcoming the limitations of manual application. This method enables localized, minimally invasive drug delivery with precise depth control, reducing the risk of perforation or unintended tissue damage^127^. The MN pen system’s ability to deliver drugs to sensitive and small areas of the eye, such as the cornea, is a significant advancement in ocular drug delivery, offering a practical solution for more precise and controlled administration^60^. The advanced version of the MN pen, as presented in this follow-up study, was developed to address the limitations of short-term drug delivery and surface-level applications of earlier systems. This detachable hybrid MN pen (d-MNP) introduces a significant improvement by allowing for the controlled, localized injection of biodegradable MN tips directly into the corneal stroma. The MN tips detach from the pen, remain embedded in the tissue, and provide sustained drug release over an extended period. This system enables precise depth-controlled drug delivery, ensuring the drug is delivered directly to the targeted area without risk of perforation. The detachable MN also solves the issue of surface obstruction often seen with patch-style MNs, making it ideal for treating corneal infections like Acanthamoeba keratitis. Overall, the d-MNP offers greater control, localized delivery, and sustained drug release, making it a highly effective option for long-term ocular treatments^42^.

In the same manner, two recent studies further advanced the MN pen technology for ocular drug delivery. Lee et al. developed a rapidly d-MNP using a porous water-soluble layer composed of PVA and PVP. This design allowed for nearly instantaneous separation of the MN tip upon insertion into the sclera, significantly reducing patient discomfort and ensuring efficient drug delivery. The optimized porosity and rapid dissolution of the sacrificial layer ensured fast detachment, while the impact insertion method enhanced tip placement, making it ideal for sustained ocular drug release^43^. Furthermore, another study applied this technology to treat infectious keratitis using a biodegradable MN pen system. This system featured a detachable MN tip loaded with antibiotics, which dissolved over several days to provide sustained drug release directly into the cornea. This single-administration approach replaced the need for multiple eye drop treatments, improving patient compliance and treatment efficacy^128^.

The MN pen technology offers clear advantages in ocular drug delivery, including precise, localized drug administration with minimal invasiveness and sustained release from biodegradable MN tips. However, challenges remain, such as ensuring consistent insertion depth and enhancing the mechanical strength of the MNs to prevent breakage. To address these issues, refinements in the pen’s insertion mechanism for better depth control and stronger yet biodegradable materials could enhance the system’s reliability and efficacy, making it an even more robust solution for ocular drug delivery.

While these advancements highlight the potential of MN pens, safety concerns must be carefully considered due to the sensitive nature of ocular tissues. One potential risk is tissue irritation or micro-damage caused by excessive insertion force, which could compromise the cornea or sclera’s structural integrity. To mitigate this, spring-loaded mechanisms in MN pens are being calibrated to deliver controlled and consistent insertion forces, ensuring safe and reliable penetration without overloading the tissues. Another critical concern is the risk of infection, particularly when reusable components are involved. The implementation of single-use, pre-sterilized MN cartridges addresses this issue by minimizing contamination risks. Additionally, incorporating biocompatible materials with anti-microbial coatings into MN designs can further enhance patient safety by reducing the likelihood of post-implantation infections. These strategies collectively aim to ensure that the clinical application of MN pens remains both effective and safe.

This section summarized key ocular MN implantation techniques, including patch-based systems, contact lens-shaped MNs, and detachable MN pens. Each method provides precise and minimally invasive drug delivery while offering sustained release for improved therapeutic outcomes. However, challenges such as maintaining consistent drug release, optimizing MN strength, and ensuring patient comfort and compliance still need to be addressed. With ongoing innovations in material engineering and device design, these systems have the potential to become more effective and reliable for long-term ocular treatments.

## Conclusion and Discussion

This review explored the use of MNs as a promising platform for controlled and sustained drug delivery to the eye. By examining the fundamental design principles, material considerations, and fabrication methods, we highlighted how these systems can offer more precise and effective treatments for ocular diseases compared to traditional methods. The versatility of MNs allows for gradual drug release over time, making them a compelling alternative for long-term therapies. However, their broader application still faces several challenges that need to be addressed for successful clinical integration. A major challenge in developing MNs for ocular drug delivery is the interdependence between key factors such as drug delivery rate, mechanical properties, and degradation or dissolution rates. These factors are not independent of one another, making it difficult to optimize each one separately. This complex relationship poses challenges in fine-tuning MNs for effective and consistent performance. Additionally, limitations in current fabrication methods hinder the miniaturization of needles to submicrometer scales, which is crucial for reducing tissue trauma and minimizing the risks of infection or delayed recovery. To address these challenges, future research directions should focus on developing multifunctional materials that integrate mechanical strength, biocompatibility, and controlled degradation properties. Innovations in stimuli-responsive materials, such as light-, pH-, or temperature-sensitive polymers, could provide enhanced control over drug release dynamics and enable on-demand therapeutic delivery. Moreover, bioinspired designs that mimic natural biological structures may pave the way for improved precision and reduced invasiveness. Advances in microfabrication techniques, including two-photon polymerization and nanoscale lithography, will be instrumental in achieving submicrometer-scale MNs, which are critical for reducing tissue damage and improving patient comfort. Additionally, the integration of MNs with wearable or implantable diagnostic systems could allow for real-time monitoring of disease progression and adaptive drug delivery, enabling highly personalized treatments. Lastly, addressing regulatory and scalability challenges will be essential for translating these innovative technologies from research labs to clinical applications. By overcoming these obstacles and leveraging emerging technologies, MNs hold the potential to revolutionize ocular drug delivery, offering safer, more effective, and personalized treatment options for patients with complex ocular diseases.
